# Adiponectin GWAS loci harboring extensive allelic heterogeneity exhibit distinct molecular consequences

**DOI:** 10.1371/journal.pgen.1009019

**Published:** 2020-09-11

**Authors:** Cassandra N. Spracklen, Apoorva K. Iyengar, Swarooparani Vadlamudi, Chelsea K. Raulerson, Anne U. Jackson, Sarah M. Brotman, Ying Wu, Maren E. Cannon, James P. Davis, Aaron T. Crain, Kevin W. Currin, Hannah J. Perrin, Narisu Narisu, Heather M. Stringham, Christian Fuchsberger, Adam E. Locke, Ryan P. Welch, Johanna K. Kuusisto, Päivi Pajukanta, Laura J. Scott, Yun Li, Francis S. Collins, Michael Boehnke, Markku Laakso, Karen L. Mohlke

**Affiliations:** 1 Department of Genetics, University of North Carolina, Chapel Hill, North Carolina, United States of America; 2 Department of Biostatistics and Epidemiology, University of Massachusetts, Amherst, Massachusetts, United States of America; 3 Department of Biostatistics and Center for Statistical Genetics, School of Public Health, University of Michigan, Ann Arbor, Michigan, United States of America; 4 National Human Genome Research Institute, National Institutes of Health, Bethesda, Maryland, United States of America; 5 Center for Biomedicine, European Academy of Bolzano/Bozen, University of Lübeck, Bolzano/Bozen, Italy; 6 McDonnell Genome Institute, Washington University School of Medicine, St. Louis, Missouri, United States of America; 7 Department of Medicine, University of Eastern Finland and Kuopio University Hospital, Kuopio, Finland; 8 Department of Human Genetics, University of California, Los Angeles, California, United States of America; 9 Department of Biostatistics, University of North Carolina, Chapel Hill, North Carolina, United States of America; New York Genome Center & Columbia University, UNITED STATES

## Abstract

Loci identified in genome-wide association studies (GWAS) can include multiple distinct association signals. We sought to identify the molecular basis of multiple association signals for adiponectin, a hormone involved in glucose regulation secreted almost exclusively from adipose tissue, identified in the Metabolic Syndrome in Men (METSIM) study. With GWAS data for 9,262 men, four loci were significantly associated with adiponectin: *ADIPOQ*, *CDH13*, *IRS1*, and *PBRM1*. We performed stepwise conditional analyses to identify distinct association signals, a subset of which are also nearly independent (lead variant pairwise r^2^<0.01). Two loci exhibited allelic heterogeneity, *ADIPOQ* and *CDH13*. Of seven association signals at the *ADIPOQ* locus, two signals colocalized with adipose tissue expression quantitative trait loci (eQTLs) for three transcripts: trait-increasing alleles at one signal were associated with increased *ADIPOQ* and *LINC02043*, while trait-increasing alleles at the other signal were associated with decreased *ADIPOQ-AS1*. In reporter assays, adiponectin-increasing alleles at two signals showed corresponding directions of effect on transcriptional activity. Putative mechanisms for the seven *ADIPOQ* signals include a missense variant (*ADIPOQ* G90S), a splice variant, a promoter variant, and four enhancer variants. Of two association signals at the *CDH13* locus, the first signal consisted of promoter variants, including the lead adipose tissue eQTL variant for *CDH13*, while a second signal included a distal intron 1 enhancer variant that showed ~2-fold allelic differences in transcriptional reporter activity. Fine-mapping and experimental validation demonstrated that multiple, distinct association signals at these loci can influence multiple transcripts through multiple molecular mechanisms.

## Author summary

Many DNA variants affect common human traits, and distinct variants can have different effects on the function or expression level of the same gene. We identified variants associated with levels of adiponectin, a hormone involved in glucose regulation. Among these variants, we specifically studied the sets of variants located near two genes, *ADIPOQ* and *CDH13*, to determine how the variants affect gene expression or function. We focused on sets of variants that can be inherited together but are not always inherited together. Of the variants associated with adiponectin and located near *ADIPOQ*, one set were also associated with higher expression levels of the protein-coding *ADIPOQ* gene and a nearby non-coding gene, a second set of variants were associated with lower levels of the *ADIPOQ-AS1* antisense gene, and additional variants changed the amino acid sequence or size of the adiponectin protein. Our examples show the benefits of identifying multiple sets of trait-associated variants in the same DNA region. These variants explain more trait variation, help identify genes that affect the trait, and guide studies of gene regulation and biological processes.

## Introduction

Genome-wide association studies (GWAS) have identified thousands of associations between genomic loci and complex traits[1, 2]. However, identification of mechanisms underlying the association signals is often complicated by numerous potential target genes and patterns of linkage disequilibrium (LD)[3]. The complexity of GWAS loci also includes the consequences of allelic heterogeneity[4]. At loci harboring allelic heterogeneity, multiple functional variants can result in multiple distinct association signals. Depending on the haplotype on which each new functional variant allele arose[5], lead variants at these signals may exhibit moderate pairwise LD or may be nearly independent (r^2^<0.01). Allelic heterogeneity can include both *cis*-regulatory and coding variants that may act alone or in combination to affect the phenotype[6]. With increasing sample sizes and use of larger imputation reference panels in array- and sequence-based GWAS, the extent of allelic heterogeneity at complex trait loci is becoming more apparent[7]. This heterogeneity creates challenges in identifying causal variants and target genes, but also leads to more comprehensive characterization of mechanisms by which GWAS variants influence the associated trait(s).

Interpretation of GWAS results includes elucidating the molecular mechanisms by which genetic variants affect gene expression or function. Early candidate gene and genome-wide association studies identified multiple coding variants associated with a complex trait within the same gene[8–10]. Other studies have reported loci where multiple regulatory variants in strong LD with each other likely affect one gene[11, 12], loci where multiple regulatory variants may affect different genes[13], and loci for which both coding and noncoding variants in the same region are independently associated with a phenotype[5, 14]. Examples of the dissection and characterization of multiple, distinct, *cis*-regulatory association signals at one locus remain few[11, 15, 16], especially when the signals are not statistically independent of each other.

To detect and characterize the molecular mechanisms at GWAS loci harboring allelic heterogeneity, we tested genetic associations with circulating plasma adiponectin levels in the Metabolic Syndrome in Men (METSIM) study. Adiponectin, encoded by the *ADIPOQ* gene, is a hormone involved in glucose regulation and secreted almost exclusively by adipose tissue[17]. We identified additional association signals within 1 Mb of significant lead GWAS variants (*P*<5x10^-8^) using stepwise conditional analyses. To identify candidate functional variant(s) and gene(s) at these association signals, we used coding annotations, colocalized adipose expression quantitative trait locus (eQTL) signals, chromatin accessibility, and epigenomic marks of transcriptional regulation, and demonstrated allelic effects of variants on regulatory activity in functional assays. Taken together, this study demonstrates that independent and distinct association signals within one locus may affect different transcripts and act via multiple distinct molecular mechanisms.

## Results

### Genetic variants associated with adiponectin levels

We conducted a genome-wide association study (GWAS) for adiponectin levels in 9,262 non-diabetic men from the METSIM study[18]. We assumed an additive genetic model and tested for association with ~16.6 million variants with MAF ≥ 0.08% (minor allele count ≥ 15). We confirmed (*P*<0.05) twelve adiponectin loci previously identified in European and East Asian individuals (S1 Table), four of which achieved genome-wide significance (rs12051272 within *CDH13* intron 1, *P* = 1.8 x 10^−68^; rs199938283 near *ADIPOQ*, *P* = 8.2 x 10^−55^; rs149689033, 630 kb upstream of *IRS1*, *P* = 3.0 x 10^−9^; and rs2276824 within an intron of *PBRM1*, *P* = 3.5 x 10^−8^; Table 1; S2 Table; S1–S5 Figs). One previously unreported locus, located 454 kb downstream of *EPHA3* (rs139269730, *P* = 4.1 x 10^−8^; S6 Fig) showed nominal genome-wide significance, although the single lead variant exhibited only moderate imputation quality (r^2^ = 0.74) and may represent a false positive. Assuming the fifth signal is a false positive, the four other lead GWAS signals explained 7.0% of variation in adiponectin levels.

**Table 1:** Loci with two or more distinct signals of adiponectin association detected using stepwise conditional analysis (locus-wide significance, *P*<1x10^-5^).

Lead variant	Chr:Position (hg19)	EA/NEA	EAF	EA count	LD (r^2^/D’)^a^	Unconditioned GWAS		Conditioned GWAS	
						Effect (SE)	*P*	Effect (SE)	*P*
*ADIPOQ*									
rs199938283 (A)	3:186,552,469	C/CAAAT	0.03	596	-	-0.653 (0.042)	8.2x10^-55^	-	-
rs4632532 (B)	3:186,551,682	C/T	0.40	11,090	0.04/0.95	-0.130 (0.015)	1.5x10^-17^	-0.193 (0.015)	1.3x10^-35^
rs16861209 (C)	3:186,563,114	A/C	0.03	532	0.00/1.00	0.435 (0.045)	2.2x10^-22^	0.311 (0.045)	4.9x10^-12^
rs73187787 (D)	3:186,701,595	T/C	0.18	3,340	0.01/0.88	0.126 (0.020)	1.9x10^-10^	0.115 (0.019)	3.8x10^-9^
rs17366653 (E)	3:186,570,816	C/T	0.002	29	0.00/1.00	-0.848 (0.195)	1.4x10^-5^	-0.983 (0.190)	2.4x10^-7^
rs17846866 (F)	3:186,570,746	G/T	0.09	1,647	0.31/0.95	-0.373 (0.026)	4.5x10^-45^	-0.161 (0.031)	2.8x10^-7^
rs62625753 (G)	3:186,572,026	A/G	0.0008	15	0.00/1.00	-0.766 (0.258)	3.0x10^-3^	-0.856 (0.251)	6.5x10^-4^
*CDH13*									
rs12051272 (A)	16:82,663,288	T/G	0.11	1,979	-	-0.430 (0.024)	1.8x10^-68^	-	-
rs4782722 (B)	16:82,672,165	T/G	0.46	10,047	0.02/0.41	0.119 (0.015)	6.8x10^-15^	0.084 (0.015)	3.2x10^-8^

Chr, chromosome; EA, effect allele; NEA, non-effect allele; EAF, effect allele frequency; GT, genotyped; LD, linkage disequilibrium; SE, standard error

^a^ LD (r^2^/D') with variant showing the strongest evidence of association at each locus.

Effect size from an additive model and corresponding to the effect allele for inverse-normal transformed adiponectin levels. P values of stepwise conditional analyses, in which we included the variant(s) with the strongest evidence of association into the regression model as a covariate(s) and continued to test for the next strongest variant until the strongest variant showed a conditional P value >1x10^-5^ or had been reported as a functional variant (*ADIPOQ* signal G, rs62625753; Gly90Ser).

At these four loci, we identified additional association signals by performing stepwise conditional analyses and, for comparison, an approximate conditional analysis. We defined “distinct” as an association signal that met a locus-wide significance threshold of *P*_*cond*_<1x10^-5^ within 1 Mb of an initial lead GWAS variant after conditioning. When the pairwise linkage disequilibrium (LD) between the signal’s lead variant and all other identified lead variants at the locus was low (r^2^<0.01), we defined the signal more specifically as “independent”. Only one association signal was identified at the *IRS1* and *PBRM1* loci (S2 Table and S4–S6 Figs). We identified additional association signals at both the *ADIPOQ* (seven total signals) and *CDH13* (two total signals) loci, described in more detail below. After adding the seven additional signals identified at the *ADIPOQ* and *CDH13* loci, the four loci explained 10.3% of the variation in adiponectin levels.

### Four independent and three distinct association signals at *ADIPOQ*

**Fig 1:**
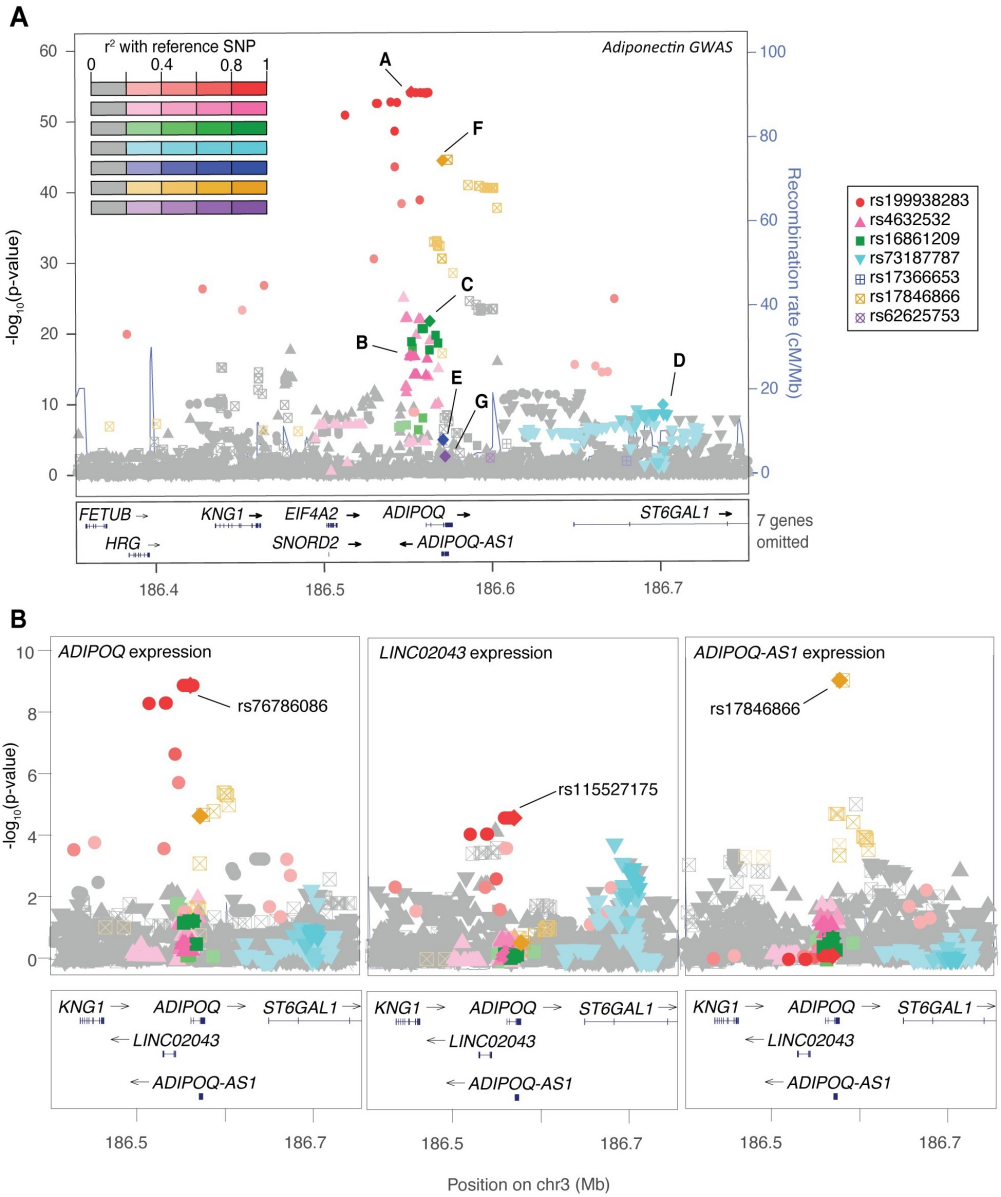
Locus with seven adiponectin GWAS signals and subcutaneous adipose eQTL signals for three transcripts. (A) Seven signals near *ADIPOQ* (labeled ‘A’-‘G’) were identified through stepwise conditional analyses for association with adiponectin levels (n = 9,262). The signals are labeled in the order in which they were identified in the stepwise process and are distinguished by both color and shape. Variants are shaded based on LD with the lead variants, shown as diamonds. (B) Two variants in perfect LD (r^2^ = 1.00) with adiponectin GWAS signal ‘A’ (rs76786086 and rs115527175) show the strongest association with expression levels of *ADIPOQ* and *LINC02043* in subcutaneous adipose tissue in 434 METSIM participants. The strongest adiponectin-associated variant from signal ‘F’ (rs17846866) also shows the strongest association with expression levels of *ADIPOQ-AS1* in subcutaneous adipose tissue in 434 METSIM participants.

*ADIPOQ* encodes the adiponectin protein measured in GWAS and is expressed almost exclusively in mature adipocytes[19]. At the *ADIPOQ* locus, stepwise conditional analysis revealed seven distinct signals associated with adiponectin levels (Table 1; S2 and S3 Tables; Fig 1; S3 and S7 Figs). After conditioning on the lead GWAS variant (rs199938283; signal ‘A’), the next strongest adiponectin-associated variant was rs4632532 (*P*_*cond*_ = 1.3x10^-35^; signal ‘B’). Variants at signal ‘B’ were previously reported by the ADIPOGen GWAS meta-analysis to have the strongest association with adiponectin levels. However, studies contributing to that analysis were imputed to the HapMap2 reference panel, which did not include any variants representing signal ‘A’[20]. Our subsequent conditional analyses identified four additional association signals (lead variants rs16861209, signal ‘C’; rs73187787, signal ‘D’; rs17366653, signal ‘E’; and rs17846866, signal ‘F’; Table 1) that each reached locus-wide significance (*P*<1x10^-5^). The next strongest signal (rs62625753, signal ‘G’) did not meet the significance threshold but is a known missense variant in exon 3 of *ADIPOQ* (G90S; Table 1) shown previously to influence adiponectin multimerization[21]. No additional coding variants within the *ADIPOQ* gene reached the locus-wide significance threshold for association with adiponectin levels (S4 Table). Altering the order by which variants were entered into the model as covariates did not alter the lead variants at any association signal, nor did selection of a different variant to represent a particular association signal or use of a different initial imputation reference panel (S5 Table). Together, the seven signals explained 5.8% of the phenotypic variance compared to 2.7% for the lead *ADIPOQ* signal alone.

To characterize the distinct signals, we examined the LD between lead variants at pairs of signals. We found the pairwise LD to be low (r^2^≤0.01) for all signal pairs except for signals ‘A’ and ‘B’ (r^2^ = 0.05) and signals ‘A’ and ‘F’ (r^2^ = 0.33), suggesting that signals ‘C’, ‘D’, ‘E’, and ‘G’ are statistically near-independent, while signals ‘A’, ‘B’, and ‘F’ are distinct, yet the trait-raising alleles are sometimes present on shared haplotypes. To determine the extent to which the three signals act independently of each other, we next performed haplotype association analyses with adiponectin levels in METSIM using the lead variants from signals ‘A’, ‘B’, and ‘F’ (S8 Fig). Compared to a reference haplotype containing the adiponectin-increasing alleles at both signals ‘A’ and ‘B’ (rs199938283-Ins and rs4632532-T), haplotypes containing at least one adiponectin-decreasing allele exhibited lower adiponectin levels (e.g. Ins-C, β = -0.193; CT, β = -0.779; S8A Fig). A similar pattern of association was observed between signals ‘A’ and ‘F’ (S8B Fig). These results confirm that within haplotypes with the same allele at signal ‘A’, variants at both signals ‘B’ and ‘F’ still contribute to variation in adiponectin levels.

We extended the haplotype association analyses with adiponectin levels to use the lead variants for all seven signals (S9 and S10 Figs). Compared to a reference haplotype containing the adiponectin-increasing alleles at each signal (rs199938283-Ins, rs4632532-T, rs16861209-A, rs73187787-T, rs17366653-T, rs17846866-T, and rs62625753-G), haplotypes containing at least one adiponectin-decreasing allele exhibited lower adiponectin levels (e.g. Ins-TACTTG, β = -0.043; Ins-TCTTTG, β = -0.278). Haplotypes containing the largest number of adiponectin-decreasing alleles exhibited the lowest levels of adiponectin when compared to the baseline haplotype. To account for potential errors in chromosome phasing, we repeated haplotype analyses using only the 2,637 METSIM participants homozygous for all seven signals; estimated effect sizes were consistent with those based on the full set of participants (S10 Fig), providing support that our estimated effect size estimates are not influenced by haplotyping errors.

### Multiple candidate transcripts at *ADIPOQ*

We next examined molecular mechanisms at the *ADIPOQ* locus, including variants in *ADIPOQ* predicted to affect the adiponectin protein (Table 2). Signals ‘E’ and ‘G’ showed the largest effects on adiponectin levels (Table 1), and both have been shown previously to alter *ADIPOQ* protein sequence. rs62625753 at signal ‘G’ encodes Gly90Ser, which decreases adiponectin multimerization[21]. Because we measured total adiponectin, we do not have data on multimers to validate the functional consequences shown previously. rs17366653 at signal ‘E’ was reported to increase the proportion of *ADIPOQ* isoform that splices out exons 2 and 3 (50 bp), leading to nonsense-mediated decay[22]. We did not have a sufficient number of study participants in the RNA-seq data that expressed the *ADIPOQ* isoform without exons 2 and 3 to examine the association between rs17366653 and expression level of the isoform.

**Table 2:** Proposed molecular consequences at multi-signal loci.

Signal	Lead variant and adiponectin-decreasing allele	Number of candidate variants (r^2^≥0.80)	Candidate functional variant(s) based on allelic differences in experimental assays	Candidate transcript and direction based on colocalized eQTL	Putative molecular consequence
***ADIPOQ***					
A	rs199938283-C	15	rs76071583	↓ *ADIPOQ*, ↓ *LINC02043*	Decreased *ADIPOQ* and *LINC02043* enhancer activity; decreased transcriptional activity (Fig 2)
B	rs4632532-C	14	-	-	Assumed decreased *ADIPOQ* enhancer activity
C	rs16861209-C	12	-	-	Assumed decreased *ADIPOQ* enhancer activity
D	rs73187787-C	7	rs13322149, rs55958900	-	Increased distal enhancer *ADIPOQ-AS1* activity; increased transcriptional activity (Fig 2)
E	rs17366653-C	1	rs17366653	-	*ADIPOQ* splice variant; reported to increase the proportion of *ADIPOQ* isoform lacking exon 2 and 3 [22]
F	rs17846866-G	2	-	↑ *ADIPOQ-AS1*	Assumed increased *ADIPOQ-AS1* enhancer activity
G	rs62625753-A	1	rs62625753 (Gly90Ser)	-	*ADIPOQ* missense variant (G90S); reported to decrease adiponectin multimerization[21]
***CDH13***					
A	rs12051272-T	8	rs12051272	↑ *CDH13*	Decreased *CDH13* proximal intron 1 promoter activity (Fig 4, S17 Fig)
B	rs4782722-G	6	rs4782722	-	*CDH13 distal* intron 1 enhancer activity; increased transcriptional activity (Fig 4, S17 Fig)

For candidate transcripts and putative molecular consequences, the directions of effect are based on the allele associated with decreased adiponectin levels in the METSIM GWAS. The putative molecular consequence column includes allele-specific functional evidence from this work and other cited publications. When no direct evidence exists for molecular consequences of a variant (*CDH13* signal ‘A’ and *ADIPOQ* signals ‘B’, ‘C’, and ‘F’), we proposed molecular functions and candidate transcript based on candidate variant locations.

To aid in the identification of candidate genes, we examined whether the association signals for adiponectin are colocalized (see Methods) with association signals for expression levels of nearby (<1 Mb) transcripts in subcutaneous adipose tissue from a subset of 434 METSIM participants (S6 Table)[23]. Adiponectin association signal ‘A’ colocalized with eQTL signals for *ADIPOQ* (rs76786086-T; β = -1.69; *P* = 1.0x10^-9^) and *LINC02043* (rs115527175-T; β = -1.19; *P* = 1.9x10^-5^), an adjacent long non-coding RNA expressed primarily in brain, adipose, testis, arterial, and splenic tissues (Table 2; S6 and S7 Tables; Fig 1B; S11 Fig). Additionally, adiponectin association signal ‘F’ colocalized with an eQTL signal for *ADIPOQ-AS1* (β = 0.77; *P* = 7.7x10^-10^), an antisense transcript overlapping portions of two distal exons of *ADIPOQ* that has been shown to post-transcriptionally regulate *ADIPOQ* (S6 and S7 Tables; Fig 1B; S11 Fig)[24]. No additional eQTL signals were detected for these three transcripts, and no other colocalized *cis*-eQTL associations were identified for these variants (S7 Table). Variants associated with lower plasma adiponectin level at signal ‘A’ were associated with lower expression levels of both *ADIPOQ* and *LINC02043*, while variants associated with lower plasma adiponectin level at signal ‘F’ were associated with higher expression level of *ADIPOQ-AS1* (S12 Fig). Expression levels of *ADIPOQ* and *ADIPOQ-AS1* are only weakly positively correlated, potentially reflecting the very low relative expression level of *ADIPOQ-AS1* and the wide distribution of expression level of *ADIPOQ*. In comparison, data from GTEx v8[25] showed different lead variants associated with expression level of *ADIPOQ* that correspond to GWAS signal ‘F’ and of *ADIPOQ-AS1* that correspond to GWAS signal ‘E’; these results may be influenced by differences in cell type composition, sex, sampling variability, and/or expression measurement/analysis (S8 Table).

We further investigated signals ‘A’ and ‘F’ at the *ADIPOQ* locus to evaluate their statistically distinct contributions to gene expression. Within the METSIM adipose eQTL data, we used further conditional analyses to investigate whether the associations between signals ‘A’ and ‘F’ and expression levels of *ADIPOQ* and *ADIPOQ-AS1* may have different molecular effects (S9 Table). The association between signal ‘A’ variant rs143784260 (LD r^2^ = 1.00 with lead signal ‘A’ variant rs199938283) and *ADIPOQ* expression level persists after conditioning on signal ‘F’ variant rs17846866 (*P*_initial_ = 1.0x10^-9^; *P*_*cond*_ = 1.3x10^-6^). Similarly, the association between signal ‘F’ variant rs17846866 and *ADIPOQ-AS1* expression level persists after conditioning on signal ‘A’ variant rs143784260 (*P*_initial_ = 7.6x10^-10^; *P*_*cond*_ = 5.6x10^-12^). The distinct contributions of each signal on transcript expression were confirmed using haplotype association analyses (S13 Fig). Compared to the reference haplotypes containing the transcript expression-increasing alleles at both *ADIPOQ* (rs143784260-C, rs17846866-T) and *ADIPOQ-AS1* (rs143784260-C, rs17846866-G), haplotypes containing at least one transcript expression-decreasing allele exhibited lower expression levels, providing further support that these two association signals have molecularly distinct effects.

### Candidate regulatory variants at *ADIPOQ*

**Fig 2:**
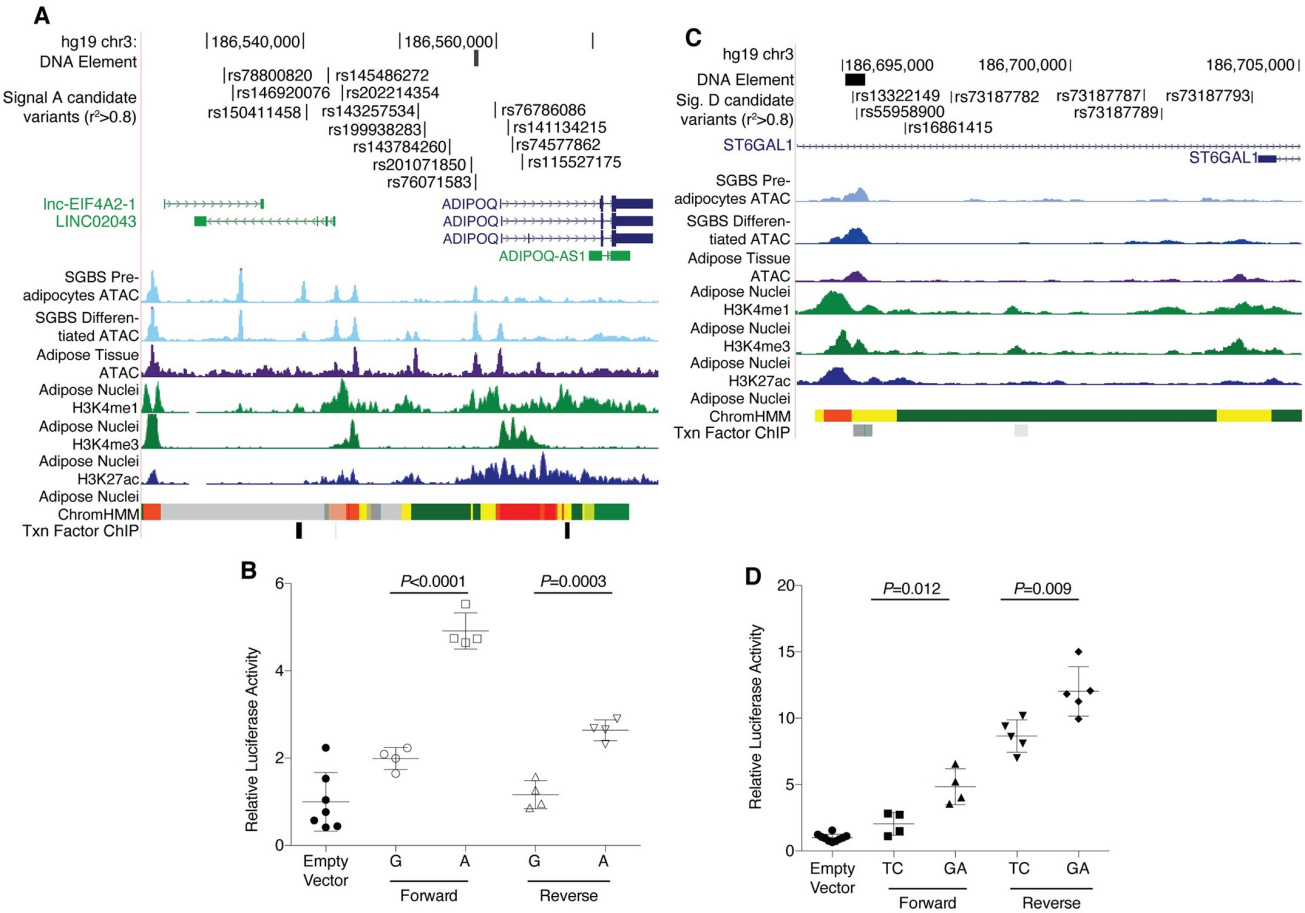
*ADIPOQ* signals ‘A’ and ‘D’ exhibit allelic differences in transcriptional activity. (A) rs199938283 and thirteen candidate variants in high pairwise LD (r^2^≥0.80) span a 25 kb region upstream of the *ADIPOQ* transcription start site and within *ADIPOQ* intron 1. (B) rs76071583-A, associated with higher adiponectin levels, showed greater transcriptional activity in both forward and reverse orientation with respect to *ADIPOQ* in 3T3L1 differentiated adipocytes compared to rs76071583-G and an “empty vector” containing a minimal promoter. (C) rs73187787 and six candidate variants in high pairwise LD (r^2^≥0.80) span a 10 kb region within *ST6GAL1* intron 2. (D) A haplotype of variant alleles rs13322149-G and rs55958900-A, associated with lower adiponectin levels, showed greater transcriptional activity in both forward and reverse orientation in 3T3L1 differentiated adipocytes compared to the TC haplotype and an “empty vector” containing a minimal promoter.

Based on the position of signal ‘A’ and ‘B’ candidate variants in accessible chromatin and chromatin marks of active enhancers in adipocytes/adipose tissue, we tested variants for allelic effects on transcriptional activity (Fig 2; S14 Fig). Of 14 candidate variants at signal ‘A’ (lead GWAS variant and variants in LD r^2^≥0.80), rs76071583, in a 462-base pair element located ~2.5 kb upstream from the *ADIPOQ* transcription start site (Fig 2A), showed the strongest allelic differences in transcriptional activity. In differentiated 3T3-L1 adipocytes, the element containing the adiponectin-increasing allele rs76071583-A showed 2.5-fold increased enhancer activity compared to the element containing the rs76071583-G allele (forward, *P*<0.0001; reverse, *P* = 0.0003; Fig 2B), suggesting that the alleles alter function of a *cis*-regulatory enhancer element. The sequence containing rs76071583-A is predicted to include a consensus core-binding motif for CEBP-α, and a ChIP-seq peak for CEBP-α also overlaps this region. In electrophoretic mobility shift assays (EMSA) using CEBP-α protein, we observed an allele-specific band for rs76071583-A (S15 Fig), suggesting that CEBP-α binds to rs76071583 to increase enhancer activity at this locus. Additional variants tested representing signals ‘A’ and ‘B’ did not exhibit allelic differences in transcriptional activity (S14 Fig). These and other variants representing signals ‘B’ and ‘C’ are interspersed in chromosomal position with variants from signal ‘A’. However, we did not observe any strong annotations of regulatory elements (S7 Fig) and the functional consequences of signals ‘B’ and ‘C’ remain to be detected.

Of candidate variants at *ADIPOQ* signal ‘D’, we examined transcriptional activity in reporter assays for a 445-base pair region spanning rs13322149 and rs55958900 located 135 kb distal to the *ADIPOQ* promoter within the first intron of *ST6GAL1* (Fig 2C). In differentiated 3T3-L1 cells, the haplotype containing both alleles associated with lower adiponectin (rs13322149-G and rs55958900-A) exhibited ≥ 2.4-fold higher enhancer activity (forward, *P* = 0.012; reverse, *P* = 0.009) compared to the haplotype containing the alleles associated with higher adiponectin (Fig 2D), suggesting that one or both variants within this haplotype alter a distal *cis*-regulatory element. While we do not have direct mechanistic evidence of a link to any transcript, the direction of effect of adiponectin decreasing alleles on transcriptional activity is consistent with an effect on *ADIPOQ-AS1*, not *ADIPOQ* or *LINC02043*. The allelic effects on transcriptional activity, distance (>100 kb) from the other signals, and direction of effect consistent with *ADIPOQ-AS1* all provide support for functional consequences molecularly distinct from the other signals.

### Two association signals at *CDH13*

**Fig 3:**
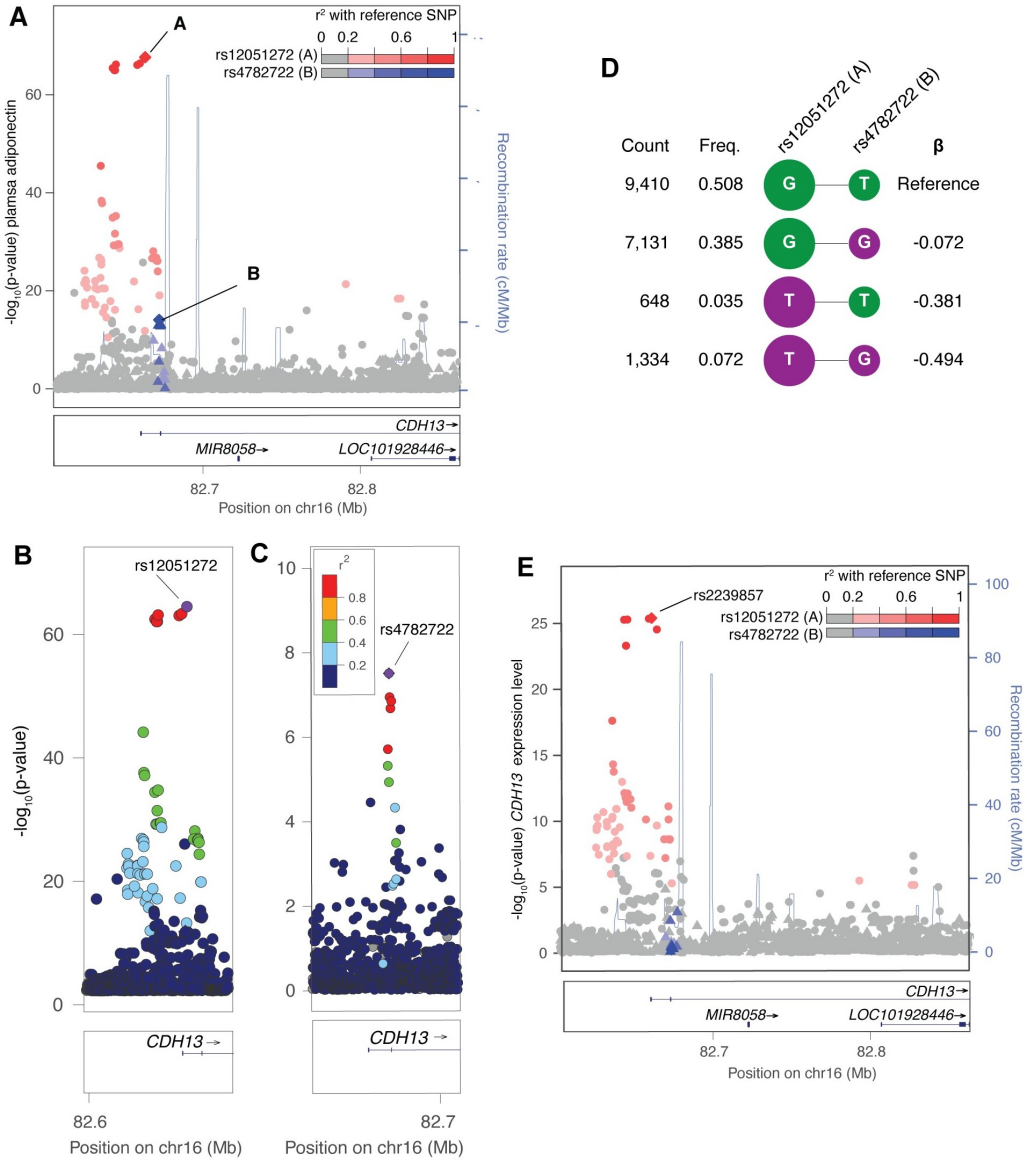
Locus with two adiponectin GWAS signals and one subcutaneous adipose eQTL signal for *CDH13*. (A) The first signal with lead variant rs12051272 (red circles, denoted as ‘A’) shows the strongest association in the adiponectin GWAS (n = 9,262). After conditioning on rs12051272, a second signal with lead variant rs4782722 (blue triangles, denoted as ‘B’) remained significant (*P*<5x10^-8^). Variants are shaded based on LD with the lead variants, shown as diamonds. (B) rs12051272 (purple diamond; signal ‘A’) shows the strongest association with adiponectin. (C) rs4782722 (purple diamond; signal ‘B’) shows the strongest association with adiponectin after conditioning on rs12051272 (signal ‘A’). (D) Haplotypes of rs12051272 and rs4782722 estimated from 9,262 METSIM participants. Alleles associated with higher adiponectin levels in single variant analyses are shown in green, alleles associated with lower adiponectin are shown in purple. Haplotype association was performed with adiponectin inverse normalized residuals after adjusting for age, age^2^, and BMI using the most frequent haplotype as the reference. (E) Variant rs2239857, in perfect LD (r^2^ = 1.00) with rs12051272, shows the strongest association with expression level of *CDH13* in subcutaneous adipose tissue in 434 METSIM participants.

Expressed in multiple cell types throughout the cardiovascular system, *CDH13* encodes the cadherin-13 receptor, a cell surface receptor for hexameric and high-molecular weight adiponectin known to influence levels of circulating adiponectin[26]. Positive feedback exists between adiponectin and the cadherin-13 receptor[27]. At the *CDH13* locus, we identified two distinct adiponectin association signals. Conditioning on the lead GWAS variant (rs12051272, signal ‘A’) revealed a second distinct signal (rs4782722, *P*_*cond*_ = 3.2x10^-8^, signal ‘B’) (Table 1; S2 Table; Fig 3A; S2 Fig). Pairwise LD between the lead variants at the two signals was low (r^2^ = 0.04). No *CDH13* coding variants reached locus-wide significance with adiponectin levels after conditioning on rs12051272 and rs4782722 (S10 Table). Compared to a reference haplotype containing the adiponectin-increasing allele at both variants (rs12051272-G and rs4782722-T), haplotypes containing at least one adiponectin-decreasing allele exhibited lower adiponectin levels (GG, β = -0.072; TT, β = -0.381; Fig 3D). Haplotypes containing both adiponectin-decreasing alleles exhibited the lowest levels of adiponectin (TG, β = -0.494) when compared to the baseline haplotype. Together, the two signals explained 3.8% of the variance in adiponectin levels compared to 3.5% for the lead *CDH13* signal alone.

### Function of candidate regulatory variants at *CDH13*

We next tested the *CDH13* signals for colocalization with subcutaneous adipose eQTL signals for nearby transcripts. The adiponectin-decreasing allele rs2239857-G, a variant in perfect LD (r^2^ = 1.0) with the lead adiponectin GWAS variant of signal ‘A’ (rs12051272; r^2^ = 1.00), is most strongly associated with increased *CDH13* expression level (β = 1.00; *P* = 4.35x10^-26^; Table 2; S6 Table; Fig 3E). While we did not find significant evidence of a second signal associated with expression level of *CDH13* or colocalizations with expression level of any other transcripts, larger eQTL studies would have more power to detect additional signals.

**Fig 4:**
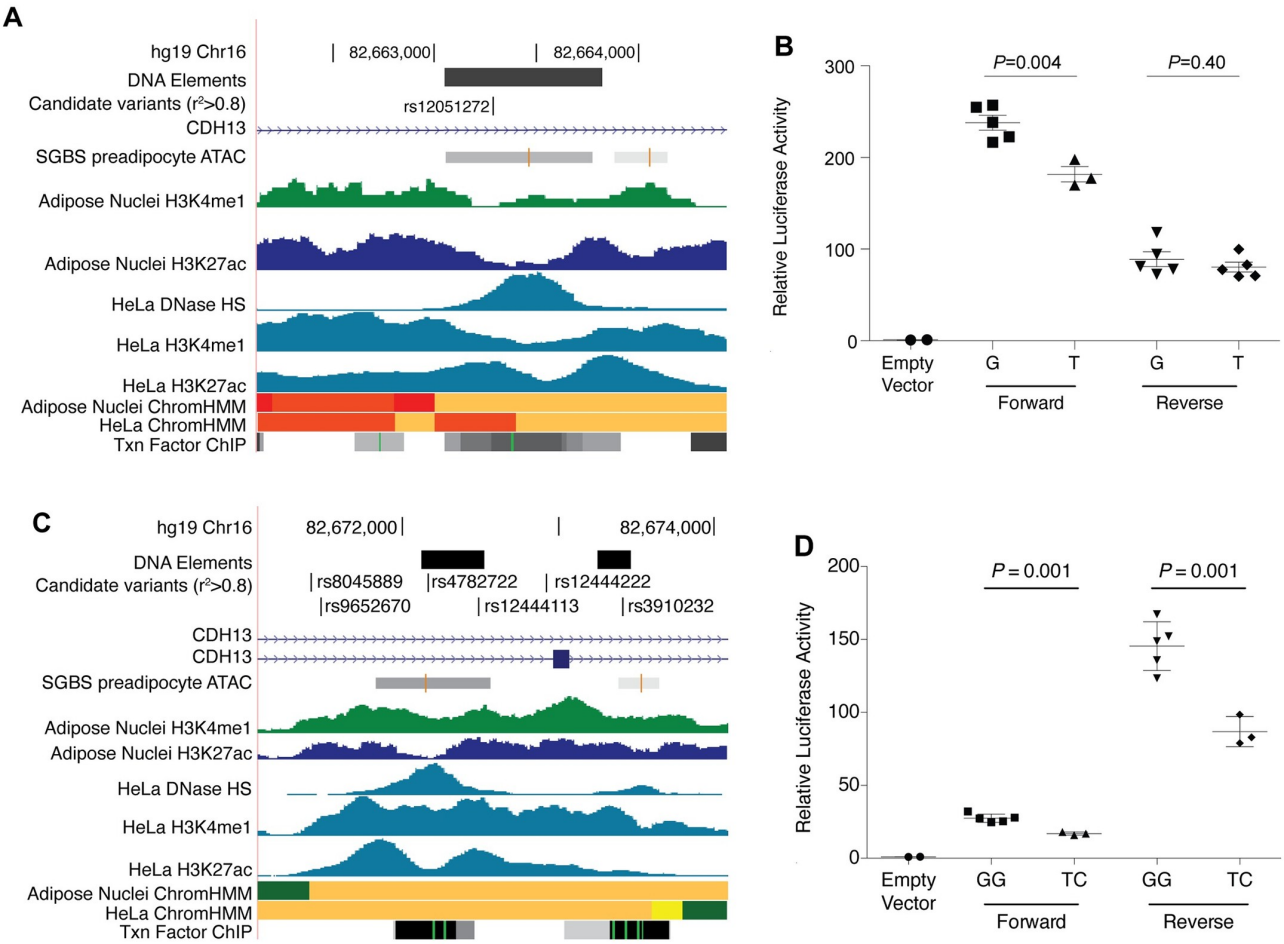
Both GWAS signals at *CDH13* (signals ‘A’ and ‘B’) exhibit allelic differences in transcriptional activity. (A) rs12051272 is located within *CDH13* intron 1. (B) rs12051272-G associated with lower adiponectin showed greater transcriptional activity in the forward orientation with respect to *CDH13* in HeLa cells compared to rs12051272-G and an “empty vector” containing a minimal promoter. (C) rs4782722 and all five candidate variants in high pairwise LD (r^2^≥0.80) span a 2.5 kb region in *CDH13* introns 1 and 2. (D) A haplotype of variant alleles rs4782722-G and rs12444113-G associated with lower adiponectin showed greater transcriptional activity in both forward and reverse orientation with respect to *CDH13* in HeLa cells compared to the TC haplotype and an “empty vector” containing a minimal promoter. Transcriptional activity results for rs3910232 are shown in S18 Fig.

To investigate whether the two statistically distinct adiponectin-association signals at *CDH13* have distinguishable consequences, we investigated potential molecular mechanisms. At both signals, no candidate variants (LD r^2^≥0.80 with rs12051272 or rs4782722) are located in coding regions. The lead signal ‘A’ variant rs12051272, is located in intron 1, ~3 kb from the *CDH13* transcription start site, in an accessible chromatin region with chromatin marks of active enhancers in multiple cell types including HeLa (Fig 4A and 4B; S16 Fig). We used HeLa cells to examine transcriptional activity in reporter assays for a 775-bp region spanning rs12051272 (Fig 4A and 4B). The adiponectin-increasing allele rs12051272-G showed an average of 1.7-fold increased enhancer activity compared to the rs12051272-T (*P* = 0.004), suggesting that rs12051272-G may be responsible for increased transcriptional activity of a target gene leading to higher adiponectin levels. The rs12051272-G allele that showed increased enhancer activity in HeLa cells was unexpectedly associated with decreased *CDH13* adipose tissue expression levels, suggesting that the effect observed in HeLa cells does not fully represent the adipose tissue signal, that *CDH13* expression level reflects feedback[27, 28], or that *CDH13* is not the target gene.

At *CDH13* signal ‘B’, we investigated three intronic variants located ~12 kb distal to the *CDH13* transcription start site in accessible chromatin regions with marks of active enhancers (rs4782722, rs12444113, and rs3910232; Fig 4C and 4D; S17 and S18 Figs). In HeLa cells, a 398-bp region containing the adiponectin-decreasing alleles of two variants (rs4782722-G and rs12444113-G) showed 1.7-fold increased enhancer activity compared to the haplotype containing the non-risk alleles (*P* = 0.0001). Additional haplotype constructs containing rs4782722-G showed a 1.4-fold increase in enhancer activity compared to haplotype constructs containing rs4782722-T (S17 Fig), suggesting that rs4782722-G is responsible for increased transcriptional activity of a target gene leading to lower adiponectin levels. Taken together, these data show that rs4782722 at *CDH13* signal ‘B’ exhibits allelic differences in transcriptional enhancer activity and suggest it functions within a distal intron 1 *cis*-regulatory element, distinct from the proximal intron 1 variants at *CDH13* signal ‘A’.

## Discussion

Here we described statistically distinct and independent complex trait association signals that act through molecularly distinct mechanisms. At the *ADIPOQ* GWAS locus, which harbors extensive allelic heterogeneity, seven association signals appear to act through two different coding mechanisms (missense variant altering protein multimerization and splice variant leading to a nonfunctional transcript) and multiple transcriptional regulatory mechanisms acting on three target transcripts, *ADIPOQ* and two long non-coding RNAs. At the *CDH13* GWAS locus, two signals associated with decreased plasma adiponectin levels likely act via proximal intron 1 promoter (signal ‘A’) and distal intron 1 enhancer (signal ‘B’) variants to affect expression levels of *CDH13*. Using molecular-based assays, we demonstrated variants at several association signals at both the *ADIPOQ* and *CDH13* loci are located in regulatory elements and exhibit significant allelic effects on transcription. Identification and molecular dissection of multiple association signals at a GWAS locus provides a more accurate measure of the explained trait variability, enabling additional molecular mechanisms and target genes to be identified, furthering our understanding of target gene regulation and the biology underlying complex traits.

Allelic heterogeneity is a common characteristic of complex traits[29], and identifying multiple signals at GWAS loci serves several purposes. First, each signal can help identify which genes influence the trait[13]. Even at loci for which an effector gene is obvious, such as *ADIPOQ* for adiponectin levels, each signal can help elucidate gene function and/or a regulatory mechanism. In addition, each association signal can account for additional trait variation and explain more trait heritability[5]. Finally, the prediction accuracy of polygenic risk scores relies on the full set of variants that affect a trait, and including variants from multiple association signals at a locus can improve prediction accuracy[30].

As GWAS sample sizes increase and more signals are identified, the intricacies of how to characterize, annotate, and/or dissect multiple signals at individual loci are becoming more apparent[4]. eQTL sample sizes are also increasing, gaining sufficient statistical power to identify and differentiate multiple signals[25]. Several methods exist for identifying multiple signals at a GWAS locus[31, 32]. We had individual-level genotype and trait data available; therefore, we performed exact stepwise conditional analyses. This method proved robust to different modifications in consistently identifying the same lead variants and order in which the signals appeared. When only summary association data are available, such as from GWAS meta-analyses, distinct signals can be detected using approximate conditional analysis with GCTA with estimated LD from a provided reference sample[32]. When we applied GCTA using the genotype information from the GWAS study samples and METSIM as a reference for LD, GCTA and stepwise conditional analyses yielded nearly identical results across all loci, with the exception of one very rare signal (*ADIPOQ* signal ‘G’). While all methods for identification of multiple association signals have limitations (e.g. computationally intensive, data availability, accurate representation for LD), identifying potentially novel additional signals can better capture the genetic architecture of GWAS loci.

Adiponectin is an adipose-tissue derived hormone that plays a central role in energy homeostasis[17]. Higher levels of adiponectin can protect from obesity, type 2 diabetes, atherosclerosis, and cardiovascular disease[33]. Interaction of adiponectin with its receptors activates signaling pathways that affect insulin signaling, nitric oxide production, adipogenesis, glucose uptake, fatty acid oxidation, lipogenesis, glycolysis, and gluconeogenesis[34]. Many loci associated with adiponectin levels are shared with other insulin resistance traits and risk of type 2 diabetes, including loci near *IRS1*, *LYPAL1*, *ARL15/FST*, *VEGFA*, *CMIP*, and *PEPD*[7]. The insulin-sensitizing, anti-diabetic, anti-atherogenic, and anti-inflammatory properties of adiponectin suggest its potential relevance as a therapeutic target for diabetes and metabolic syndrome[34].

Given the obvious biological link between the *ADIPOQ* gene and circulating plasma adiponectin levels, both candidate gene studies and GWAS have previously identified pronounced associations between variants in and near *ADIPOQ* and adiponectin levels[35–37]. Previous GWAS meta-analyses have consistently reported the strongest associations for variants in high LD (r^2^≥0.80) with signal ‘B’ from this study[20, 38]. However, these meta-analyses used genotype data imputed to the relatively sparse HapMap2 reference panel that does not include representative variants from signals ‘A’, ‘D’, ‘E’, ‘F’, or ‘G’. Similar to our observation, another study using the denser UK10K reference panel identified stronger adiponectin association for variants within signal ‘A’[39]. Experimental studies have also identified variants that functionally affect adiponectin levels, including rs17366653 (signal ‘E’) and rs62625753 (signal ‘G’) and other coding variants not polymorphic or available in this study[22, 40, 41]. Our results suggest that many statistically independent (signals ‘C’, ‘D’, ‘E’, ‘G’) and distinct (signals ‘A’, ‘B’, and ‘F’) association signals exist that act via distinct molecular mechanisms to affect adiponectin levels. Association analyses in larger studies will likely detect additional signals and may reveal additional potential molecular mechanisms.

*ADIPOQ* association signal ‘A’ was colocalized with eQTL signals for *ADIPOQ* and *LINC02043*, while *ADIPOQ* association signal ‘F’ was colocalized with an eQTL for *ADIPOQ-AS1*, suggesting that the functional variant(s) at each signal may regulate expression of a non-coding transcript. Very little is known about *LINC02043*, which was very lowly expressed, but long non-coding RNAs can regulate nearby genes in *cis* through mechanisms such as transcriptional initiation, splicing, mRNA stability, translation, and the regulation of epigenetic modifications[42, 43]. The alleles associated with decreased plasma adiponectin levels at *ADIPOQ* signal ‘A’ are associated with decreased expression of both *ADIPOQ* and *LINC02043*, suggesting that *LINC02043* may play a role in coordinating the regulatory activity of *ADIPOQ*, although *LINC02043* may simply be affected by nearby enhancers but not influence adiponectin levels. Previous work has suggested that *ADIPOQ-AS1* plays a post-transcriptional role in the regulation of *ADIPOQ*, inhibiting adipogenesis through the formation of an *ADIPOQ-AS1/ADIPOQ* mRNA complex to suppress the translation of *ADIPOQ* mRNA[24, 44]. In addition, overexpression of *ADIPOQ-AS1* was shown to inhibit the differentiation of preadipocytes, suggesting *ADIPOQ-AS1* plays a crucial role in adipogenesis[24]. The directions of association we observe between variants at *ADIPOQ* signal ‘F’ with both decreased plasma adiponectin levels and increased expression level of *ADIPOQ-AS1* are consistent with this role.

Complex loci can include multiple association signals that are located close in physical position yet are independent or distinct. Some GWAS loci are relatively straightforward, with a single association signal, functional variant, and mechanism. We demonstrate that multiple signals within a locus may also be functionally examined separately from each other, even when they are not statistically independent, and that multiple signals can exhibit distinct effects through multiple mechanisms and multiple transcripts. Full characterization of GWAS loci with regards to signal identification, fine-mapping, and functional annotation can provide insight into the genetic architecture of the loci and the corresponding traits.

## Methods

### Ethics statement

The METSIM study was approved by the Ethics Committee of the University of Eastern Finland and Kuopio University Hospital in Kuopio, Finland and carried out in accordance with the Helsinki Declaration. Written informed consent was obtained from all participants.

### Study participants

The study included 9,262 non-diabetic men from the population-based METSIM study (mean age 57±7 SD years and BMI 26.8±3.8 kg/m^2^)[18].

### Adiponectin measurements

All METSIM participants participated in a one-day outpatient visit to the Clinical Research Unit at the University of Eastern Finland for data collection, which included an interview for their medical history and a blood sample following a 12-hour fast. Plasma adiponectin was measured using the Human Adiponectin ELISA kit (LINCO Research, St. Charles, MO, USA).

### Genotyping and imputation

We genotyped the METSIM participants using the HumanOmniExpress-12v1_C BeadChip and Infinium HumanExome-12 v1.0 BeadChip[45]. Genotypes were imputed using the HRC reference panel[46], and separately using the GoT2D reference panel[45], a dense reference panel of >19 million variants (SNPs, in-dels, and large deletions) based on the whole genome sequencing of 2,657 Europeans individuals from Germany, Sweden, Finland, and Britain. Following quality control[45], we tested 16,607,452 variants for association with adiponectin.

### Single-variant analysis

We tested for adiponectin association using the imputed dosages for all variants with summed minor allele count dosage >1 assuming an additive model of inheritance and accounting for cryptic relatedness using the EMMAX linear mixed model approach implemented in EPACTS[47]. Circulating adiponectin levels were adjusted for BMI, age, and age^2^, and then residuals were inverse normalized. Sensitivity analyses were also performed adjusting for age, age^2^, and fat mass percentage; results were similar (S5 Table).

### Conditional analyses

At loci that exhibited genome-wide evidence of association (*P*<5x10^-8^), we performed a series of stepwise conditional analyses by adding the most strongly associated variant into the regression model as a covariate and testing for association with all remaining variants within 1 Mb of the initial lead GWAS variant at each locus. At loci with genome-wide evidence of association, we set a less stringent, locus-wide significance threshold of *P*<1x10^-5^ to define additional signals based on an approximate 5,000 tests performed at a locus.[7] We performed stepwise conditional analyses until the strongest association variant showed a conditional *P*-value > 1x10^-5^ or had no annotation evidence that suggested a functional role. Sensitivity analyses were also performed altering the order at which signals were included in the stepwise conditional analysis and altering which variant was selected as the representative for each association signal; results were similar.

We also used Genome-wide Complex Trait Analysis[32] (GCTA) approximate regression models to identify multiple association signals at locus-wide significance (*P*<1x10^-5^) using genotype data from 10,197 METSIM participants as the LD reference panel. Haplotype analyses were performed using Haplostats in R (https://cran.r-project.org/web/packages/haplo.stats/index.html). We created regional association plots using LocusZoom[48]. Unless otherwise noted, all LD estimates were calculated based on the METSIM data from 10,197 individuals; reported allele frequencies were calculated based on the 9,262 individuals included in this analysis.

We evaluated the proportion of variance explained by a single variant or any given locus by including the variant or a set of variants into a linear regression model with all covariates used in association analysis and calculating the R^2^ for the full model. We performed these analyses using SAS version 9.4 (SAS Institute, Cary, NC, USA).

### Subcutaneous adipose eQTLs

To aid in the identification of candidate genes at the most strongly associated signals, we examined whether any of the variants associated with adiponectin were also associated with expression of nearby transcripts in subcutaneous adipose tissue. The expression quantitative trait locus (eQTL) data were generated from a subset of 434 METSIM participants with RNA-sequencing data[23]. A false discovery rate (FDR) <1% was used to identify *cis*-eQTLs. We also performed stepwise conditional analyses at each colocalized locus, conditioning stepwise on the variant most strongly associated with gene expression, to identify additional eQTL signals for each transcript. GWAS and eQTL signals were considered to be colocalized when (a) the GWAS variant and the variant most strongly associated with expression level of the corresponding transcript exhibit high pairwise LD (r^2^≥0.80), and (b) the association of the eQTL signal became insignificant (FDR>1%) after conditioning on the GWAS variant. For colocalization analyses with *ADIPOQ* signal ‘A’, we used proxy variant rs143784260 to represent rs199938283 (r^2^ = 1.00; calculated in METSIM), which was not available in the *cis*-eQTL dataset.

### Variant annotation

To establish a set of candidate functional variants at the *ADIPOQ* and *CDH13* loci, we used transcriptional regulatory chromatin marks (accessible chromatin and histone modification states) to predict which variants may affect the transcription of the nearby genes. We compiled regulatory elements from ENCODE[49], ChromHMM[50], Roadmap Epigenomics Project[51] data available through the UCSC Genome Browser[52], and accessible chromatin data from adipose tissue, preadipocytes, and adipocytes[53, 54]. We defined candidate variants as those located within accessible chromatin peaks, Roadmap ChromHMM chromatin states of enhancers and promoters, and chromatin-immunoprecipitation sequencing (ChIP-seq) peaks of histone modifications H3K4me1, H3K4me3, and H3K27ac, and transcription factor binding sites in adipose-derived mesenchymal stem cells and adipocyte nuclei (*ADIPOQ* locus) and HeLa cells (*CDH13* locus only).

### Cell culture

Cell lines were selected based on expression patterns of nearby genes. Because *ADIPOQ* is expressed almost exclusively in mature adipocytes[25], we used 3T3-L1 cells differentiated into mature adipocytes for experiments with variants at the *ADIPOQ* locus. *CDH13* is expressed throughout most of the tissues in the body, including vascular cells and adipose tissue[25]. Because variants at the *CDH13* locus were observed to be located in accessible chromatin regions in HeLa cells, we performed experiments with these variants in HeLa cells. 3T3-L1 cells (ATCC, CL-173) were cultured and differentiated as described in the ATCC protocol. HeLa cervical cancer cells (ATCC CCL-2) were cultured in DMEM/Ham’s F-12 (Corning) supplemented with 10% FBS. All cell lines were maintained at 37°C with 5% CO_2_.

### Transcriptional reporter assays

We designed oligonucleotide primers (S11 Table) with KpnI and XhoI restriction sites. Using Q5 High-Fidelity DNA Polymerase (New England BioLabs), we PCR-amplified fragments surrounding each variant from DNA of individuals homozygous for each allele and cloned the PCR product into the multiple cloning site of a minimal promoter firefly luciferase reporter vector (pGL4.23, Promega) in both orientations with respect to the reporter gene. For *CDH13* signal ‘B’, rs4782722 in the 398 bp construct was altered to create missing haplotypes using the QuikChange II Site Directed Mutagenesis Kit (Agilent Technologies). Three to five isolated clones for each allele for each orientation were sequenced to verify genotype. Cells were seeded with approximately 2×10^5^ (3T3-L1) or 6×10^5^ (HeLa) cells per well in 24-well plates. Each clone was co-transfected with *Renilla* luciferase vector (phRL-TK, Promega) in duplicate (HeLa) or triplicate (differentiated 3T3-L1) using Lipofectamine 2000 (Life Technologies) according to manufacturer’s recommendations. After 48 hours transfection, we lysed cells with passive lysis buffer (Promega) and measured luciferase activity using the Dual-Luciferase Reporter Assay System (Promega). We normalized firefly luciferase readings to *Renilla* luciferase readings and normalized all readings to the average of two empty pGL4.23 vector transfections. We used 2-sided Student’s *t*-tests to compare allelic differences in firefly luciferase activity. All transfections were repeated independently and yielded comparable results.

### Electrophoretic mobility shift assay (EMSA)

We designed 17-bp biotin-labeled and unlabeled complementary oligonucleotide probes for both rs76071583 alleles (S11 Table). As previously described[55], using the LightShift Chemiluminescent EMSA Kit (Thermo Fisher Scientific), 300 femtomole of biotin-labeled double-stranded oligos were incubated with 450 ng recombinant CEBP-a protein (Creative Biomart CEBPA-153H). To test the specificity of the protein complexes to the allele, 50x excess unlabeled probes were added to the binding reactions. We repeated EMSA experiments on independent days and obtained consistent results.

## Supporting information

S1 Fig Manhattan plot for the genome-wide association study of plasma adiponectin levels from 9,262 participants in the Metabolic Syndrome in Men (METSIM) study. Plasma adiponectin levels were inverse normal transformed following adjustment for age, age^2^, and BMI. -log_10_(*P*-values) of association results are plotted against hg19 genomic coordinates. Loci achieving genome-wide significance are labeled and include *IRS1*, *PBRM1*, *ADIPOQ*, and *CDH13*. A potentially novel adiponectin locus on chromosome 3, *EPHA3*, was also identified, but the only genome-wide significant variant, rs139269730, had moderate imputation quality (r^2^ = 0.74) and may represent a false positive (see S6 Fig), thus, this locus was excluded from further analyses.
(TIF)

S2 Fig Adiponectin locus near *CDH13* exhibits two association signals. (A) The purple diamond represents rs12051272, the strongest associated variant in the initial unconditioned analysis of plasma adiponectin. Other variants are colored based on LD with the lead variant within the METSIM subjects. (B) After conditioning on rs12051272, an additional signal, rs4782722, persisted. (C) No additional association signals persisted after conditioning on rs12051272 and rs4782722.
(TIF)

S3 Fig Adiponectin locus near *ADIPOQ* exhibits seven association signals. (A) The purple diamond represents rs199938283, the strongest associated variant in the initial unconditioned analysis of plasma adiponectin at this locus. Other variants are colored based on LD with the lead variant within the METSIM subjects. (B) After conditioning on rs199938283, the lead adiponectin-associated variant is rs4632532. (C) After conditioning on rs199938283 and rs4632532, an additional signal with lead variant rs16861209, persisted. (D) After conditioning on rs199938283, rs4632532, and rs16861209, an additional signal with lead variant rs73187787, persisted. (E) After conditioning on rs199938283, rs4632532, rs16861209, and rs73187787, an additional signal with lead variant rs17366653, persisted. (F) After conditioning on rs199938283, rs4632532, rs16861209, rs73187787, and rs17366653, an additional signal with lead variant rs17846866, persisted. (G) After conditioning on rs199938283, rs4632532, rs16861209, rs73187787, rs17366653, and 17846866, an additional signal, rs62625753, persisted. (H) No additional association signals persisted after conditioning on rs199938283, rs4632532, rs16861209, rs73187787, rs17366653, rs17846866, and rs62625753. Y-axis scale varies between panels to best show the results of each analysis.
(TIF)

S4 Fig Adiponectin locus near *IRS1* exhibits one association signal. (A) The purple diamond represents rs149689033, the strongest associated variant at this locus. Other variants are colored based on LD with the lead variant within the METSIM subjects. (B) After conditioning on rs149689033, no additional association signals persisted.
(TIF)

S5 Fig Adiponectin locus near *PBRM1* exhibits one association signal. (A) The purple diamond represents rs2276824, the strongest associated variant at this locus. Other variants are colored based on LD with the lead variant within the METSIM subjects. (B) After conditioning on rs2276824, no additional association signals persisted.
(TIF)

S6 Fig Adiponectin locus near *EPHA3* exhibits one association signal. (A) The purple diamond represents rs139269730, the strongest associated variant at this locus (rs139269730-T: β = -0.243, SE = 0.044, *P* = 4.1x10^-8^, effect allele frequency = 0.74). Other variants are colored based on LD with the lead variant within the METSIM subjects. (B) After conditioning on rs139269730, no additional association signals persisted. The ~3 Mb gap in variants observed in the plots represents the centromere of chromosome 3. The only genome-wide significant variant had moderate imputation quality (r^2^ = 0.74) and may represent a false positive, thus, this locus was excluded from further analyses.
(TIF)

S7 Fig Location of candidate variants at *ADIPOQ*. The lead adiponectin-associated variants at *ADIPOQ* signals A-G (excluding signal ‘D’) and variants in high LD (r^2^≥0.80) are shown. Variants representing signal ‘A’ are shown in red, signal ‘B’ in magenta, signal ‘C’ in green, signal ‘E’ in navy, signal ‘F’ in brown, and signal ‘G’ in dark purple.
(TIF)

S8 Fig Adiponectin haplotype association analysis for *ADIPOQ* lead variants that are not independent (LD r^2^>.01). Haplotypes were constructed in 9,262 participants from the METSIM study using the lead variant of each association signal using HaploStats. ‘Count’ indicates the number of estimated haplotypes. (A) Haplotypes for association signals ‘A’ and ‘B’. (B) Haplotypes for association signals ‘A’ and ‘F’. Haplotypes with the same allele for any given signal (e.g. signal ‘A’) show different effect sizes (betas) consistent with their single variant results for alleles at the other signals (e.g. B, F). Alleles associated with lower adiponectin are shown in purple while alleles associated with higher adiponectin are shown in green. Haplotype association was performed with adiponectin inverse normalized residuals after adjusting for age, age^2^, and BMI using the haplotype containing the adiponectin-increasing alleles at all seven signals as the reference.
(TIF)

S9 Fig Adiponectin haplotype association analysis at *ADIPOQ* in the 9,262 participants from the METSIM study. A) Haplotype of all adiponectin-increasing alleles used as the baseline reference. B) Haplotype with the largest sample size used as the baseline reference (same as the baseline reference haplotype used in S10 Fig). Haplotypes with the same allele for any given signal (e.g. signal ‘A’) show different effect sizes (betas) consistent with their single variant results for alleles at the other signals (e.g. B, C, etc). Haplotypes were constructed with the lead variant of each association signal using HaploStats. ‘Count’ indicates the number of estimated haplotypes. Alleles associated with lower adiponectin are shown in purple while alleles associated with higher adiponectin are shown in green. Haplotype association was performed with adiponectin inverse normalized residuals after adjusting for age, age^2^, and BMI using the haplotype containing the adiponectin-increasing alleles at all seven signals as the reference. The dashed line divides common haplotypes from rare (haplotype frequency <0.001.
(TIF)

S10 Fig Adiponectin haplotype association analysis in the 5,274 participants from the METSIM study who are homozygous for all seven *ADIPOQ* association signals. Haplotypes were constructed with the lead variant of each association signal using HaploStats. ‘Count’ indicates the number of estimated haplotypes. Alleles associated with lower adiponectin are shown in purple while alleles associated with higher adiponectin are shown in green. Haplotype association was performed with adiponectin inverse normalized residuals after adjusting for age, age^2^, and BMI using the most prevalent haplotype as the reference. The order of haplotype effect sizes is consistent with the order of haplotype effect sizes for all 9,262 study participants shown in S9 Fig.
(TIF)

S11 Fig Distribution of expression levels for *ADIPOQ* (A), *ADIPOQ-AS1* (B), and *LNC02043* (C) in subcutaneous adipose tissue from 434 males from the METSIM study. Expression levels are stratified by genotype for the variant most strongly associated with each transcript (eQTL): rs143784260 (A and C) and rs17846866 (B). The lead adiponectin GWAS variant for signal ‘A’, rs199938283, was not available in the eQTL dataset; however, the lead eQTL variant rs143784260 is in perfect LD (r^2^ = 1.00). For each transcript, expression levels are presented in transcript per million (TPMs) on the left and normalized TPMs after PEER correction on the right.
(TIF)

S12 Fig Comparison of expression levels of *ADIPOQ*, *ADIPOQ-AS1*, and *LNC02043*. Correlations between expression levels of *ADIPOQ* and *ADIPOQ-AS1* (A) or *ADIPOQ* and *LNC02043* (B) are shown using transcripts per million (TPM) values in the left plot and normalized TPMs after correction for PEER factors on the right. Pearson correlation (*R*^*2*^) values and corresponding *P*-values are displayed for each plot. (C) Before normalization, the distribution of TPM values is much wider for *ADIPOQ* (approximate range 0–2500) than for either *ADIPOQ-AS1* (approximate range 0–27) or *LNC02043* (0–3.5). The upper right plot in (C) is a zoomed-in version of the plot on the left. Following PEER factor correction and inverse normal transformation, TPM values are evenly distributed between the three transcripts (bottom right plot); these values were used in eQTL analyses.
(TIF)

S13 Fig Haplotype association analysis for expression levels of three transcripts in 434 participants from the METSIM study. Haplotypes are shown for expression of (A) *ADIPOQ*, (B) *ADIPOQ-AS1*, and (C) *LNC02043*. Expression levels are shown in transcripts per million (TPMs) for plots on the left and normalized TPMs after PEER correction on the right. Haplotypes with the same allele for any given signal (e.g. signal ‘A’) show different effect sizes (betas) consistent with their single variant results for alleles at the other signals (i.e. ‘F’). Haplotypes were constructed with the lead variant of each association signal using HaploStats. ‘Count’ indicates the number of estimated haplotypes. Alleles associated with lower expression are shown in purple, while alleles associated with higher expression are shown in green.
(TIF)

S14 Fig Additional candidate variants from ADIPOQ signals ‘A’ and ‘B’ did not exhibit allelic differences in transcriptional activity in differentiated 3T3L1 adipocytes. (A) rs150411458 for signal ‘A’. (B) A haplotype of four variants rs1648705 (signal ‘B’), rs4632532 (signal ‘B’), rs1648707 (signal ‘B’), and rs143257534 (signal ‘A’). (C) A haplotype of two variants rs4632532 (signal ‘B’) and rs1648707 (signal ‘B’).
(TIF)

S15 Fig CEBP-α binds to rs76071583 to increase enhancer activity. Electromobility shift assay (EMSA) with biotin-labeled probes containing the A or G allele of rs76071583 and purified CEBPA protein show an allele-specific band (lane 6 versus lane 2) that is competed away more effectively by 50-fold excess of unlabeled probe containing the A allele (lane 7) than the G allele (lane 8). An arrow points to an allele-specific protein complex binding to the A allele.
(TIF)

S16 Fig *CDH13* signal A candidate variants and replicated experiments. (A) Positions of the lead adiponectin-associated GWAS variant in this study, rs12051272, and seven proxy variants (r^2^≥0.80; “EUR Candidates”) are shown. (B) Adiponectin genome-wide association results from the METSIM study shown with East Asian LD (1000 Genomes Phase 3) and METSIM LD. The reported lead variant associated with adiponectin in East Asians is rs4783244 is in strong LD with rs12051272 in East Asian populations (r^2^ = 0.92) but not in Finns(r^2^ = 0.05). (C) Results from replicated transcriptional activity experiments of *CDH13* signal ‘A’ show rs12051272-G is consistently associated with greater transcriptional activity in both the forward and reverse orientations with respect to *CDH13* in HeLa cells compared to rs12051272-T and an “empty vector” containing a minimal promoter. Using a linear regression model to analyze data from all three transcriptional activity experiments provides further support that rs12051272-G is associated with greater transcriptional activity in both the forward (*P* = 0.02) and reverse (*P* = 0.004) directions.
(TIF)

S17 Fig The second signal at *CDH13* (signal ‘B’) exhibits allelic differences in transcriptional activity. (A) rs4782722 and all five candidate variants in high pairwise LD (r^2^≥0.80) span a 2.5 kb region in *CDH13* introns 1 and 2. (B) At *CDH13* signal ‘B’, additional haplotypes of rs4782722 and rs12444113 created by site-directed mutagenesis of rs4782722 implicate rs4782722 as a regulatory variant. Transcriptional reporter assays of a regulatory region spanning rs4782722 and rs12444113 in HeLa cells show that haplotypes containing rs4782722-G (**G**G and **G**C) exhibited greater transcriptional activity than haplotypes containing rs4782722-T (**T**G and **T**C) in the forward and reverse orientations. Each dot represents transcriptional activity of an independent experimental clone.
(TIF)

S18 Fig rs3910232 at *CDH13* signal ‘B’ does not exhibit allelic differences in transcriptional activity in HeLa cells. rs3910232 is a proxy of rs4782722 but does not appear to contribute to transcriptional activity differences at this locus.
(TIF)

S1 Table METSIM association results (P<0.05) for previously reported adiponectin-associated variants. (XLSX)

S2 Table GWAS signals of adiponectin association at locus-wide significance using sequential conditional analysis. (XLSX)

S3 Table METSIM linkage disequilibrium values between identified signals at *ADIPOQ*. (XLSX)

S4 Table Adiponectin-association results in METSIM at *ADIPOQ* coding variants. (XLSX)

S5 Table Sensitivity analysis comparing adiponectin-associated GWAS loci across reference panels and adjustments at locus-wide significance using sequential conditional analysis. (XLSX)

S6 Table Colocalization of adiponectin GWAS signals and subcutaneous adipose *cis*-eQTLs (RNA-seq, n = 434). (XLSX)

S7 Table *ADIPOQ* GWAS variant eQTL associations in METSIM subcutaneous adipose (n = 434) for transcripts surrounding ADIPOQ. (XLSX)

S8 Table Interrogation of subcutaneous adipose eQTLs for adiponectin-associated variants at the *ADIPOQ* locus in GTEx (July 2, 2019). (XLSX)

S9 Table Reciprocal conditional analysis of adiponectin GWAS signals ‘A’ and ‘F’ at *ADIPOQ* and cis-eQTLs for *ADIPOQ* and *ADIPOQ-AS1* (RNA-seq, n = 434). (XLSX)

S10 Table Adiponectin association results in METSIM at *CDH13* coding variants. (XLSX)

S11 Table Primer sequences used in functional assays. (XLSX)

## References

[1] MacArthur J, Bowler E, Cerezo M, Gil L, Hall P, Hastings E, et al The new NHGRI-EBI Catalog of published genome-wide association studies (GWAS Catalog). Nucleic acids research. 2017;45(D1):D896–d901. Epub 2016/12/03. 10.1093/nar/gkw1133 . 27899670 PMC5210590

[2] Visscher PM, Wray NR, Zhang Q, Sklar P, McCarthy MI, Brown MA, et al 10 Years of GWAS Discovery: Biology, Function, and Translation. American journal of human genetics. 2017;101(1):5–22. Epub 2017/07/08. 10.1016/j.ajhg.2017.06.005 . 28686856 PMC5501872

[3] Gallagher MD, Chen-Plotkin AS. The Post-GWAS Era: From Association to Function. American journal of human genetics. 2018;102(5):717–30. Epub 2018/05/05. 10.1016/j.ajhg.2018.04.002 . 29727686 PMC5986732

[4] Cannon ME, Mohlke KL. Deciphering the Emerging Complexities of Molecular Mechanisms at GWAS Loci. American journal of human genetics. 2018;103(5):637–53. Epub 2018/11/06. 10.1016/j.ajhg.2018.10.001 . 30388398 PMC6218604

[5] Wood AR, Hernandez DG, Nalls MA, Yaghootkar H, Gibbs JR, Harries LW, et al Allelic heterogeneity and more detailed analyses of known loci explain additional phenotypic variation and reveal complex patterns of association. Human molecular genetics. 2011;20(20):4082–92. Epub 2011/07/30. 10.1093/hmg/ddr328 . 21798870 PMC3177649

[6] Castel SE, Cervera A, Mohammadi P, Aguet F, Reverter F, Wolman A, et al Modified penetrance of coding variants by cis-regulatory variation contributes to disease risk. Nature genetics. 2018;50(9):1327–34. Epub 2018/08/22. 10.1038/s41588-018-0192-y . 30127527 PMC6119105

[7] Mahajan A, Taliun D, Thurner M, Robertson NR, Torres JM, Rayner NW, et al Fine-mapping type 2 diabetes loci to single-variant resolution using high-density imputation and islet-specific epigenome maps. Nature genetics. 2018;50(11):1505–13. Epub 2018/10/10. 10.1038/s41588-018-0241-6 . 30297969 PMC6287706

[8] Rivas MA, Beaudoin M, Gardet A, Stevens C, Sharma Y, Zhang CK, et al Deep resequencing of GWAS loci identifies independent rare variants associated with inflammatory bowel disease. Nature genetics. 2011;43(11):1066–73. Epub 2011/10/11. 10.1038/ng.952 . 21983784 PMC3378381

[9] Hugot JP, Chamaillard M, Zouali H, Lesage S, Cezard JP, Belaiche J, et al Association of NOD2 leucine-rich repeat variants with susceptibility to Crohn’s disease. Nature. 2001;411(6837):599–603. Epub 2001/06/01. 10.1038/35079107 . 11385576

[10] Nejentsev S, Walker N, Riches D, Egholm M, Todd JA. Rare variants of IFIH1, a gene implicated in antiviral responses, protect against type 1 diabetes. Science (New York, NY). 2009;324(5925):387–9. Epub 2009/03/07. 10.1126/science.1167728 . 19264985 PMC2707798

[11] Chatterjee S, Kapoor A, Akiyama JA, Auer DR, Lee D, Gabriel S, et al Enhancer Variants Synergistically Drive Dysfunction of a Gene Regulatory Network In Hirschsprung Disease. Cell. 2016;167(2):355–68.e10. Epub 2016/10/04. 10.1016/j.cell.2016.09.005 . 27693352 PMC5113733

[12] Roman TS, Marvelle AF, Fogarty MP, Vadlamudi S, Gonzalez AJ, Buchkovich ML, et al Multiple Hepatic Regulatory Variants at the GALNT2 GWAS Locus Associated with High-Density Lipoprotein Cholesterol. American journal of human genetics. 2015;97(6):801–15. Epub 2015/12/08. 10.1016/j.ajhg.2015.10.016 . 26637976 PMC4678431

[13] Spracklen CN, Horikoshi M, Kim YJ, Lin K, Bragg F, Moon S, et al Identification of type 2 diabetes loci in 433,540 East Asian individuals. Nature. 2020;582(7811):240–5. Epub 2020/06/06. 10.1038/s41586-020-2263-3 . 32499647 PMC7292783

[14] Willer CJ, Sanna S, Jackson AU, Scuteri A, Bonnycastle LL, Clarke R, et al Newly identified loci that influence lipid concentrations and risk of coronary artery disease. Nature genetics. 2008;40(2):161–9. Epub 2008/01/15. 10.1038/ng.76 . 18193043 PMC5206900

[15] Bojesen SE, Pooley KA, Johnatty SE, Beesley J, Michailidou K, Tyrer JP, et al Multiple independent variants at the TERT locus are associated with telomere length and risks of breast and ovarian cancer. Nature genetics. 2013;45(4):371–84, 84e1–2. Epub 2013/03/29. 10.1038/ng.2566 . 23535731 PMC3670748

[16] Dunning AM, Michailidou K, Kuchenbaecker KB, Thompson D, French JD, Beesley J, et al Breast cancer risk variants at 6q25 display different phenotype associations and regulate ESR1, RMND1 and CCDC170. Nature genetics. 2016;48(4):374–86. Epub 2016/03/02. 10.1038/ng.3521 . 26928228 PMC4938803

[17] Woodward L, Akoumianakis I, Antoniades C. Unravelling the adiponectin paradox: novel roles of adiponectin in the regulation of cardiovascular disease. British journal of pharmacology. 2017;174(22):4007–20. Epub 2016/10/21. 10.1111/bph.13619 . 27629236 PMC5659989

[18] Laakso M, Kuusisto J, Stancakova A, Kuulasmaa T, Pajukanta P, Lusis AJ, et al METabolic Syndrome In Men (METSIM) Study: a resource for studies of metabolic and cardiovascular diseases. Journal of lipid research. 2017 Epub 2017/01/26. 10.1194/jlr.O072629 PMC5335588 28119442

[19] Ghaben AL, Scherer PE. Adipogenesis and metabolic health. Nature reviews Molecular cell biology. 2019;20(4):242–58. Epub 2019/01/06. 10.1038/s41580-018-0093-z . 30610207

[20] Dastani Z, Hivert MF, Timpson N, Perry JR, Yuan X, Scott RA, et al Novel loci for adiponectin levels and their influence on type 2 diabetes and metabolic traits: a multi-ethnic meta-analysis of 45,891 individuals. PLoS genetics. 2012;8(3):e1002607 Epub 2012/04/06. 10.1371/journal.pgen.1002607 22479202 PMC3315470

[21] Jungtrakoon P, Plengvidhya N, Tangjittipokin W, Chimnaronk S, Salaemae W, Chongjaroen N, et al Novel adiponectin variants identified in type 2 diabetic patients reveal multimerization and secretion defects. PloS one. 2011;6(10):e26792 Epub 2011/11/03. 10.1371/journal.pone.0026792 . 22046359 PMC3202584

[22] Lee BP, Lloyd-Laney HO, Locke JM, McCulloch LJ, Knight B, Yaghootkar H, et al Functional characterisation of ADIPOQ variants using individuals recruited by genotype. Molecular and cellular endocrinology. 2016;428:49–57. Epub 2016/03/22. 10.1016/j.mce.2016.03.020 . 26996131

[23] Raulerson CK, Ko A, Kidd JC, Currin KW, Brotman SM, Cannon ME, et al Adipose Tissue Gene Expression Associations Reveal Hundreds of Candidate Genes for Cardiometabolic Traits. American journal of human genetics. 2019;105(4):773–87. Epub 2019/10/01. 31564431 10.1016/j.ajhg.2019.09.001 PMC6817527

[24] Cai R, Sun Y, Qimuge N, Wang G, Wang Y, Chu G, et al Adiponectin AS lncRNA inhibits adipogenesis by transferring from nucleus to cytoplasm and attenuating Adiponectin mRNA translation. Biochimica et biophysica acta Molecular and cell biology of lipids. 2018;1863(4):420–32. Epub 2018/02/08. 10.1016/j.bbalip.2018.01.005 . 29414510

[25] Gamazon ER, Segre AV, van de Bunt M, Wen X, Xi HS, Hormozdiari F, et al Using an atlas of gene regulation across 44 human tissues to inform complex disease- and trait-associated variation. Nature genetics. 2018;50(7):956–67. Epub 2018/06/30. 10.1038/s41588-018-0154-4 . 29955180 PMC6248311

[26] Hug C, Wang J, Ahmad NS, Bogan JS, Tsao TS, Lodish HF. T-cadherin is a receptor for hexameric and high-molecular-weight forms of Acrp30/adiponectin. Proceedings of the National Academy of Sciences of the United States of America. 2004;101(28):10308–13. Epub 2004/06/24. 10.1073/pnas.0403382101 . 15210937 PMC478568

[27] Matsuda K, Fujishima Y, Maeda N, Mori T, Hirata A, Sekimoto R, et al Positive feedback regulation between adiponectin and T-cadherin impacts adiponectin levels in tissue and plasma of male mice. Endocrinology. 2015;156(3):934–46. Epub 2014/12/17. 10.1210/en.2014-1618 . 25514086 PMC4330303

[28] Goddeke S, Knebel B, Fahlbusch P, Horbelt T, Poschmann G, van de Velde F, et al CDH13 abundance interferes with adipocyte differentiation and is a novel biomarker for adipose tissue health. International journal of obesity (2005). 2018;42(5):1039–50. Epub 2018/02/23. 10.1038/s41366-018-0022-4 . 29467502

[29] Hormozdiari F, Zhu A, Kichaev G, Ju CJ, Segrè AV, Joo JWJ, et al Widespread Allelic Heterogeneity in Complex Traits. American journal of human genetics. 2017;100(5):789–802. Epub 2017/05/06. 10.1016/j.ajhg.2017.04.005 . 28475861 PMC5420356

[30] Peterson RE, Kuchenbaecker K, Walters RK, Chen CY, Popejoy AB, Periyasamy S, et al Genome-wide Association Studies in Ancestrally Diverse Populations: Opportunities, Methods, Pitfalls, and Recommendations. Cell. 2019;179(3):589–603. Epub 2019/10/15. 10.1016/j.cell.2019.08.051 . 31607513 PMC6939869

[31] Valdar W, Sabourin J, Nobel A, Holmes CC. Reprioritizing genetic associations in hit regions using LASSO-based resample model averaging. Genetic epidemiology. 2012;36(5):451–62. Epub 2012/05/03. 10.1002/gepi.21639 . 22549815 PMC3470705

[32] Yang J, Lee SH, Goddard ME, Visscher PM. GCTA: a tool for genome-wide complex trait analysis. American journal of human genetics. 2011;88(1):76–82. Epub 2010/12/21. 10.1016/j.ajhg.2010.11.011 21167468 PMC3014363

[33] Wang X, Bao W, Liu J, Ouyang YY, Wang D, Rong S, et al Inflammatory markers and risk of type 2 diabetes: a systematic review and meta-analysis. Diabetes care. 2013;36(1):166–75. Epub 2012/12/25. 10.2337/dc12-0702 . 23264288 PMC3526249

[34] Achari AE, Jain SK. Adiponectin, a Therapeutic Target for Obesity, Diabetes, and Endothelial Dysfunction. Int J Mol Sci. 2017;18(6). Epub 2017/06/22. 10.3390/ijms18061321 . 28635626 PMC5486142

[35] Jee SH, Sull JW, Lee JE, Shin C, Park J, Kimm H, et al Adiponectin concentrations: a genome-wide association study. American journal of human genetics. 2010;87(4):545–52. Epub 2010/10/05. 10.1016/j.ajhg.2010.09.004 . 20887962 PMC2948810

[36] Menzaghi C, Trischitta V, Doria A. Genetic influences of adiponectin on insulin resistance, type 2 diabetes, and cardiovascular disease. Diabetes. 2007;56(5):1198–209. Epub 2007/02/17. 10.2337/db06-0506 . 17303804

[37] Hivert MF, Manning AK, McAteer JB, Florez JC, Dupuis J, Fox CS, et al Common variants in the adiponectin gene (ADIPOQ) associated with plasma adiponectin levels, type 2 diabetes, and diabetes-related quantitative traits: the Framingham Offspring Study. Diabetes. 2008;57(12):3353–9. Epub 2008/09/09. 10.2337/db08-0700 . 18776141 PMC2584143

[38] Wu Y, Gao H, Li H, Tabara Y, Nakatochi M, Chiu YF, et al A meta-analysis of genome-wide association studies for adiponectin levels in East Asians identifies a novel locus near WDR11-FGFR2. Hum Mol Genet. 2014;23(4):1108–19. Epub 2013/10/10. 10.1093/hmg/ddt488 . 24105470 PMC3900106

[39] Walter K, Min JL, Huang J, Crooks L, Memari Y, McCarthy S, et al The UK10K project identifies rare variants in health and disease. Nature. 2015;526(7571):82–90. Epub 2015/09/15. 10.1038/nature14962 . 26367797 PMC4773891

[40] Waki H, Yamauchi T, Kamon J, Ito Y, Uchida S, Kita S, et al Impaired multimerization of human adiponectin mutants associated with diabetes. Molecular structure and multimer formation of adiponectin. The Journal of biological chemistry. 2003;278(41):40352–63. Epub 2003/07/25. 10.1074/jbc.M300365200 . 12878598

[41] Kottyan LC, Woo JG, Keddache M, Banach W, Crimmins NA, Dolan LM, et al Novel variations in the adiponectin gene (ADIPOQ) may affect distribution of oligomeric complexes. SpringerPlus. 2012;1(1):66 Epub 2013/02/12. 10.1186/2193-1801-1-66 . 23396303 PMC3565092

[42] Font-Cunill B, Arnes L, Ferrer J, Sussel L, Beucher A. Long Non-coding RNAs as Local Regulators of Pancreatic Islet Transcription Factor Genes. Frontiers in genetics. 2018;9:524 Epub 2018/11/22. 10.3389/fgene.2018.00524 . 30459811 PMC6232259

[43] Kopp F, Mendell JT. Functional Classification and Experimental Dissection of Long Noncoding RNAs. Cell. 2018;172(3):393–407. Epub 2018/01/27. 10.1016/j.cell.2018.01.011 . 29373828 PMC5978744

[44] Pang WJ, Lin LG, Xiong Y, Wei N, Wang Y, Shen QW, et al Knockdown of PU.1 AS lncRNA inhibits adipogenesis through enhancing PU.1 mRNA translation. Journal of cellular biochemistry. 2013;114(11):2500–12. Epub 2013/06/12. 10.1002/jcb.24595 . 23749759

[45] Davis JP, Huyghe JR, Locke AE, Jackson AU, Sim X, Stringham HM, et al Common, low-frequency, and rare genetic variants associated with lipoprotein subclasses and triglyceride measures in Finnish men from the METSIM study. PLoS genetics. 2017;13(10):e1007079 Epub 2017/10/31. 10.1371/journal.pgen.1007079 . 29084231 PMC5679656

[46] McCarthy S, Das S, Kretzschmar W, Delaneau O, Wood AR, Teumer A, et al A reference panel of 64,976 haplotypes for genotype imputation. Nature genetics. 2016 Epub 2016/08/23. 10.1038/ng.3643 . 27548312 PMC5388176

[47] Kang HM, Sul JH, Service SK, Zaitlen NA, Kong SY, Freimer NB, et al Variance component model to account for sample structure in genome-wide association studies. Nature genetics. 2010;42(4):348–54. Epub 2010/03/09. 10.1038/ng.548 . 20208533 PMC3092069

[48] Pruim RJ, Welch RP, Sanna S, Teslovich TM, Chines PS, Gliedt TP, et al LocusZoom: regional visualization of genome-wide association scan results. Bioinformatics (Oxford, England). 2010;26(18):2336–7. Epub 2010/07/17. 10.1093/bioinformatics/btq419 . 20634204 PMC2935401

[49] An integrated encyclopedia of DNA elements in the human genome. Nature. 2012;489(7414):57–74. Epub 2012/09/08. 10.1038/nature11247 . 22955616 PMC3439153

[50] Ernst J, Kheradpour P, Mikkelsen TS, Shoresh N, Ward LD, Epstein CB, et al Mapping and analysis of chromatin state dynamics in nine human cell types. Nature. 2011;473(7345):43–9. Epub 2011/03/29. 10.1038/nature09906 . 21441907 PMC3088773

[51] Kundaje A, Meuleman W, Ernst J, Bilenky M, Yen A, Heravi-Moussavi A, et al Integrative analysis of 111 reference human epigenomes. Nature. 2015;518(7539):317–30. Epub 2015/02/20. 10.1038/nature14248 . 25693563 PMC4530010

[52] Kent WJ, Sugnet CW, Furey TS, Roskin KM, Pringle TH, Zahler AM, et al The human genome browser at UCSC. Genome research. 2002;12(6):996–1006. Epub 2002/06/05. 10.1101/gr.229102 . 12045153 PMC186604

[53] Allum F, Shao X, Guenard F, Simon MM, Busche S, Caron M, et al Characterization of functional methylomes by next-generation capture sequencing identifies novel disease-associated variants. Nat Commun. 2015;6:7211 Epub 2015/05/30. 10.1038/ncomms8211 . 26021296 PMC4544751

[54] Cannon ME, Currin KW, Young KL, Perrin HJ, Vadlamudi S, Safi A, et al Open Chromatin Profiling in Adipose Tissue Marks Genomic Regions with Functional Roles in Cardiometabolic Traits. G3 (Bethesda, Md). 2019 Epub 2019/06/13. 10.1534/g3.119.400294 . 31186305 PMC6686932

[55] Fogarty MP, Cannon ME, Vadlamudi S, Gaulton KJ, Mohlke KL. Identification of a regulatory variant that binds FOXA1 and FOXA2 at the CDC123/CAMK1D type 2 diabetes GWAS locus. PLoS genetics. 2014;10(9):e1004633 Epub 2014/09/12. 10.1371/journal.pgen.1004633 . 25211022 PMC4161327

