# Supplementary figures and images for: Adiponectin GWAS loci harboring extensive allelic heterogeneity exhibit distinct molecular consequences
Source: PLoS Genet. 2020 Sep 11;16(9):e1009019. doi: 10.1371/journal.pgen.1009019 (PMC7511027; doi:10.1371/journal.pgen.1009019)

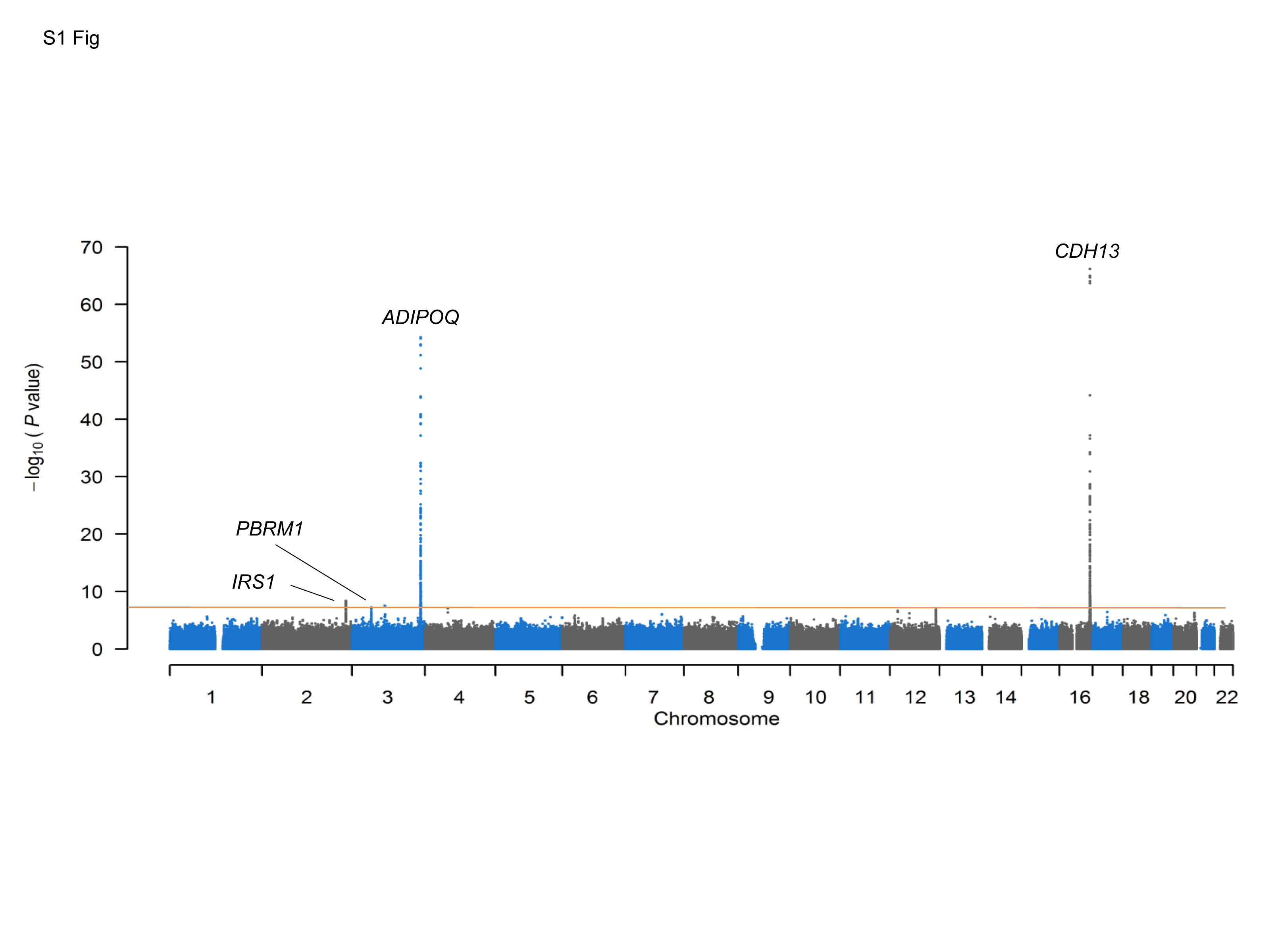

Supplement: S1 Fig — Plasma adiponectin levels were inverse normal transformed following adjustment for age, age2, and BMI. -log10(P-values) of association results are plotted against hg19 genomic coordinates. Loci achieving genome-wide significance are labeled and include IRS1, PBRM1, ADIPOQ, and CDH13. A potentially novel adiponectin locus on chromosome 3, EPHA3, was also identified, but the only genome-wide significant variant, rs139269730, had moderate imputation quality (r2 = 0.74) and may represent a false positive (see S6 Fig), thus, this locus was excluded from further analyses. (TIF) [file pgen.1009019.s001.tif]

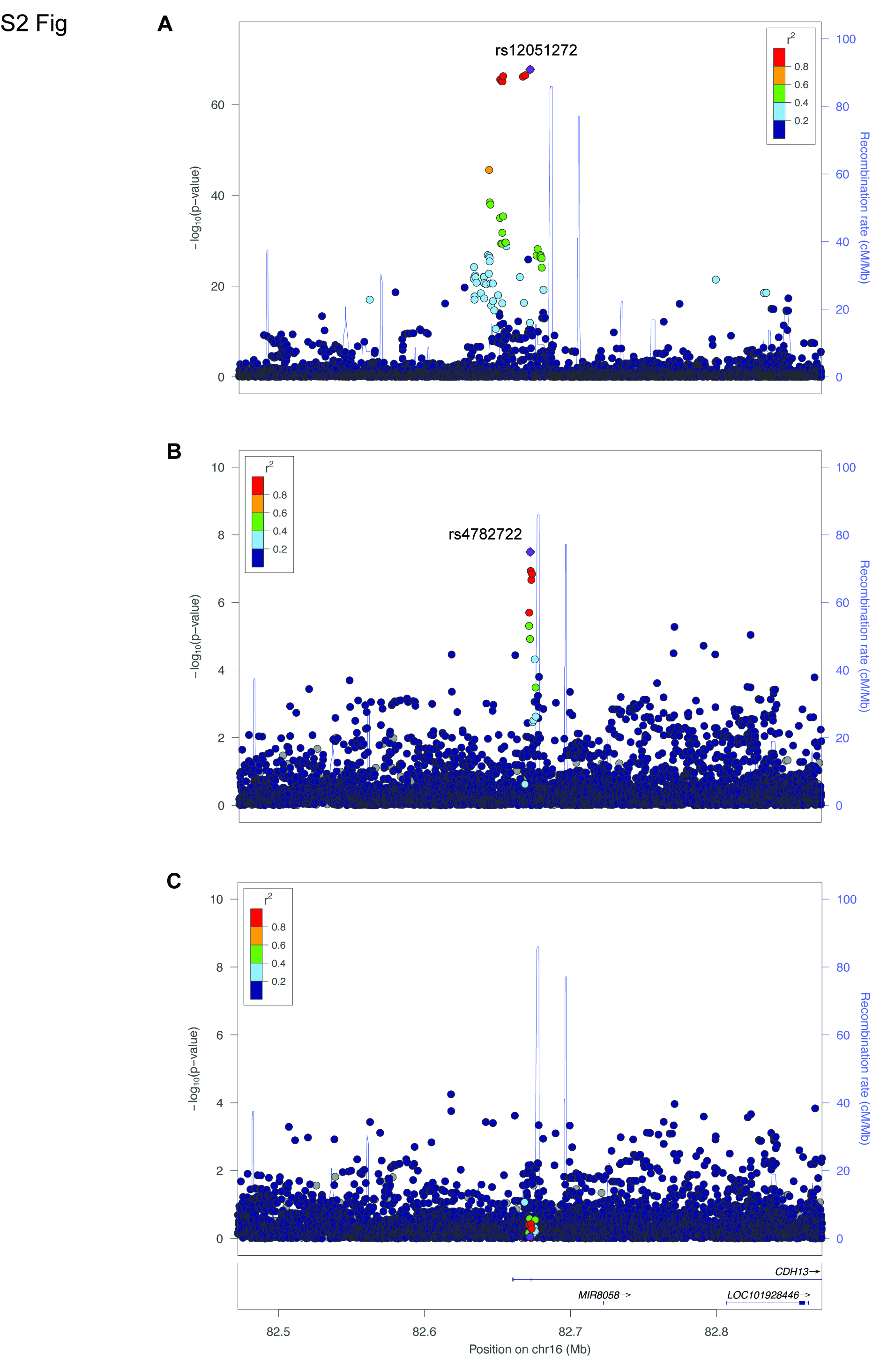

Supplement: S2 Fig — (A) The purple diamond represents rs12051272, the strongest associated variant in the initial unconditioned analysis of plasma adiponectin. Other variants are colored based on LD with the lead variant within the METSIM subjects. (B) After conditioning on rs12051272, an additional signal, rs4782722, persisted. (C) No additional association signals persisted after conditioning on rs12051272 and rs4782722. (TIF) [file pgen.1009019.s002.tif]

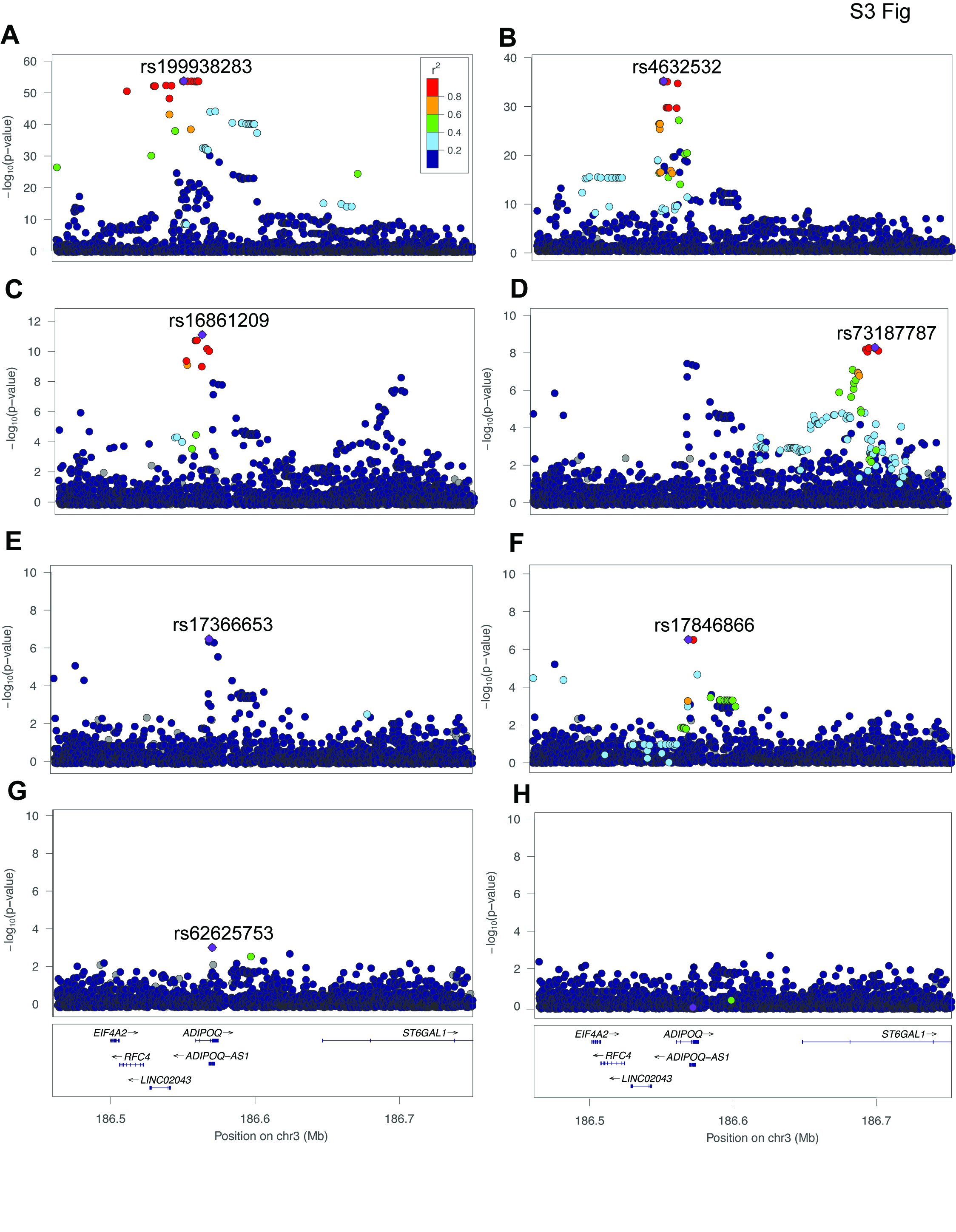

Supplement: S3 Fig — (A) The purple diamond represents rs199938283, the strongest associated variant in the initial unconditioned analysis of plasma adiponectin at this locus. Other variants are colored based on LD with the lead variant within the METSIM subjects. (B) After conditioning on rs199938283, the lead adiponectin-associated variant is rs4632532. (C) After conditioning on rs199938283 and rs4632532, an additional signal with lead variant rs16861209, persisted. (D) After conditioning on rs199938283, rs4632532, and rs16861209, an additional signal with lead variant rs73187787, persisted. (E) After conditioning on rs199938283, rs4632532, rs16861209, and rs73187787, an additional signal with lead variant rs17366653, persisted. (F) After conditioning on rs199938283, rs4632532, rs16861209, rs73187787, and rs17366653, an additional signal with lead variant rs17846866, persisted. (G) After conditioning on rs199938283, rs4632532, rs16861209, rs73187787, rs17366653, and 17846866, an additional signal, rs62625753, persisted. (H) No additional association signals persisted after conditioning on rs199938283, rs4632532, rs16861209, rs73187787, rs17366653, rs17846866, and rs62625753. Y-axis scale varies between panels to best show the results of each analysis. (TIF) [file pgen.1009019.s003.tif]

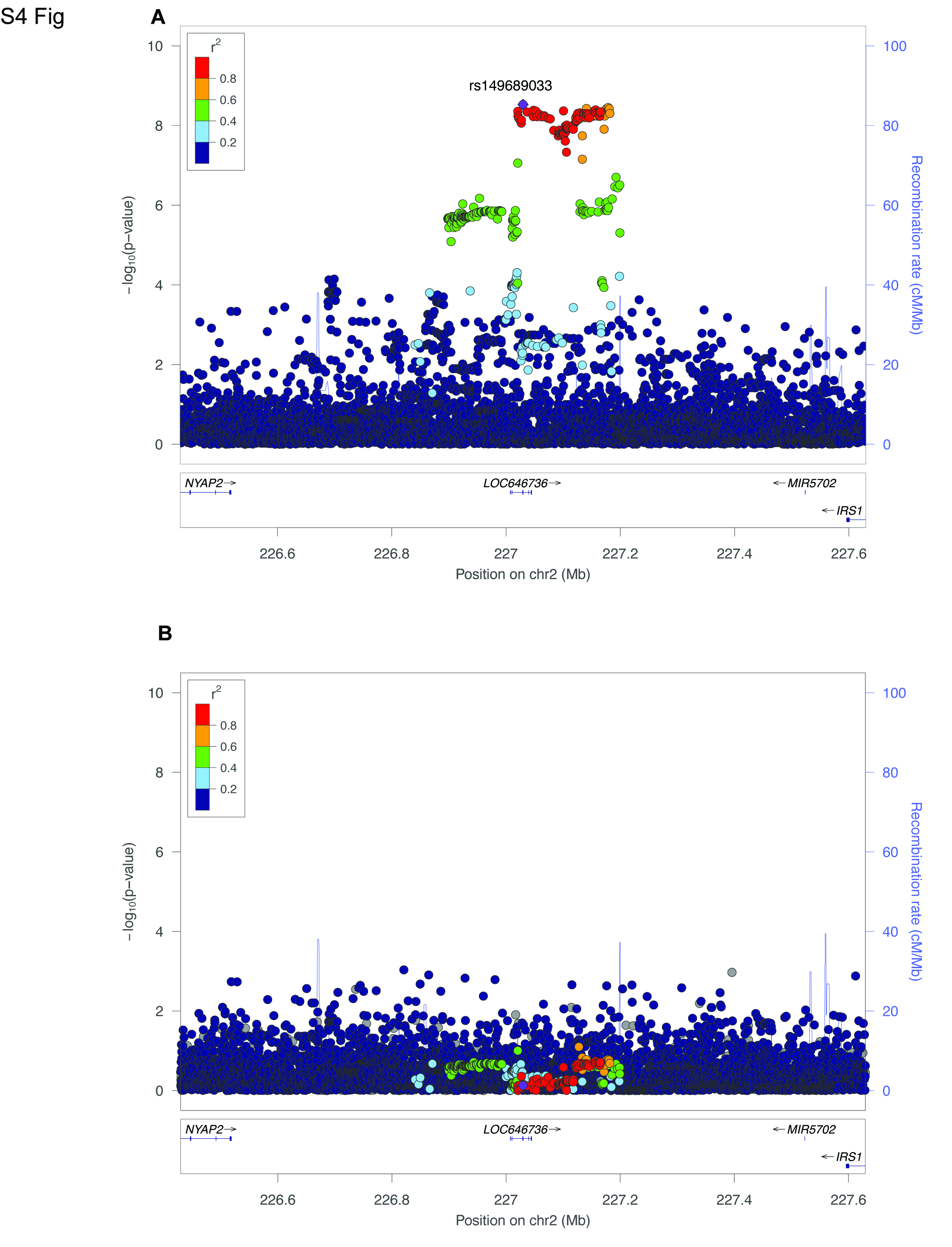

Supplement: S4 Fig — (A) The purple diamond represents rs149689033, the strongest associated variant at this locus. Other variants are colored based on LD with the lead variant within the METSIM subjects. (B) After conditioning on rs149689033, no additional association signals persisted. (TIF) [file pgen.1009019.s004.tif]

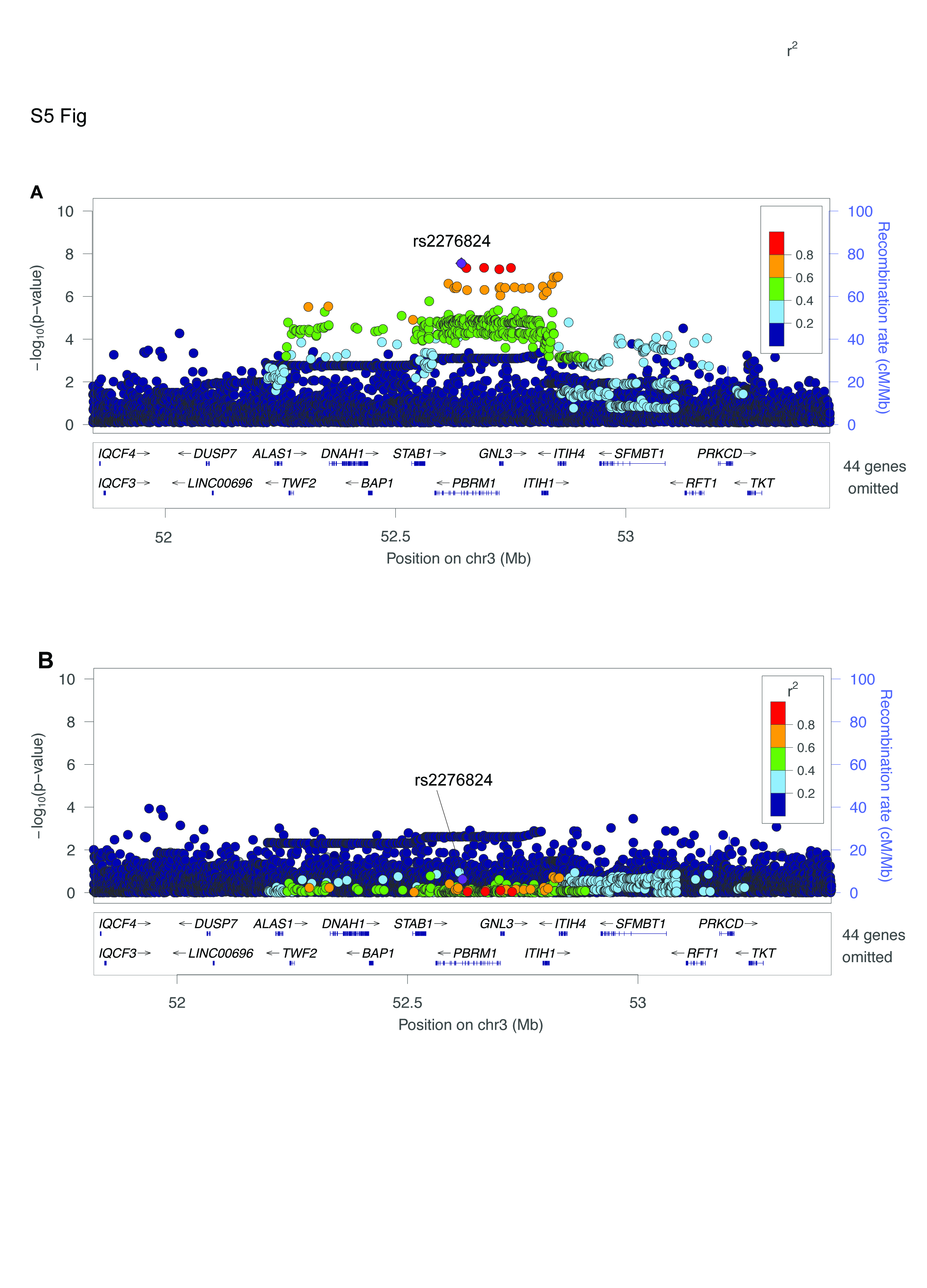

Supplement: S5 Fig — (A) The purple diamond represents rs2276824, the strongest associated variant at this locus. Other variants are colored based on LD with the lead variant within the METSIM subjects. (B) After conditioning on rs2276824, no additional association signals persisted. (TIF) [file pgen.1009019.s005.tif]

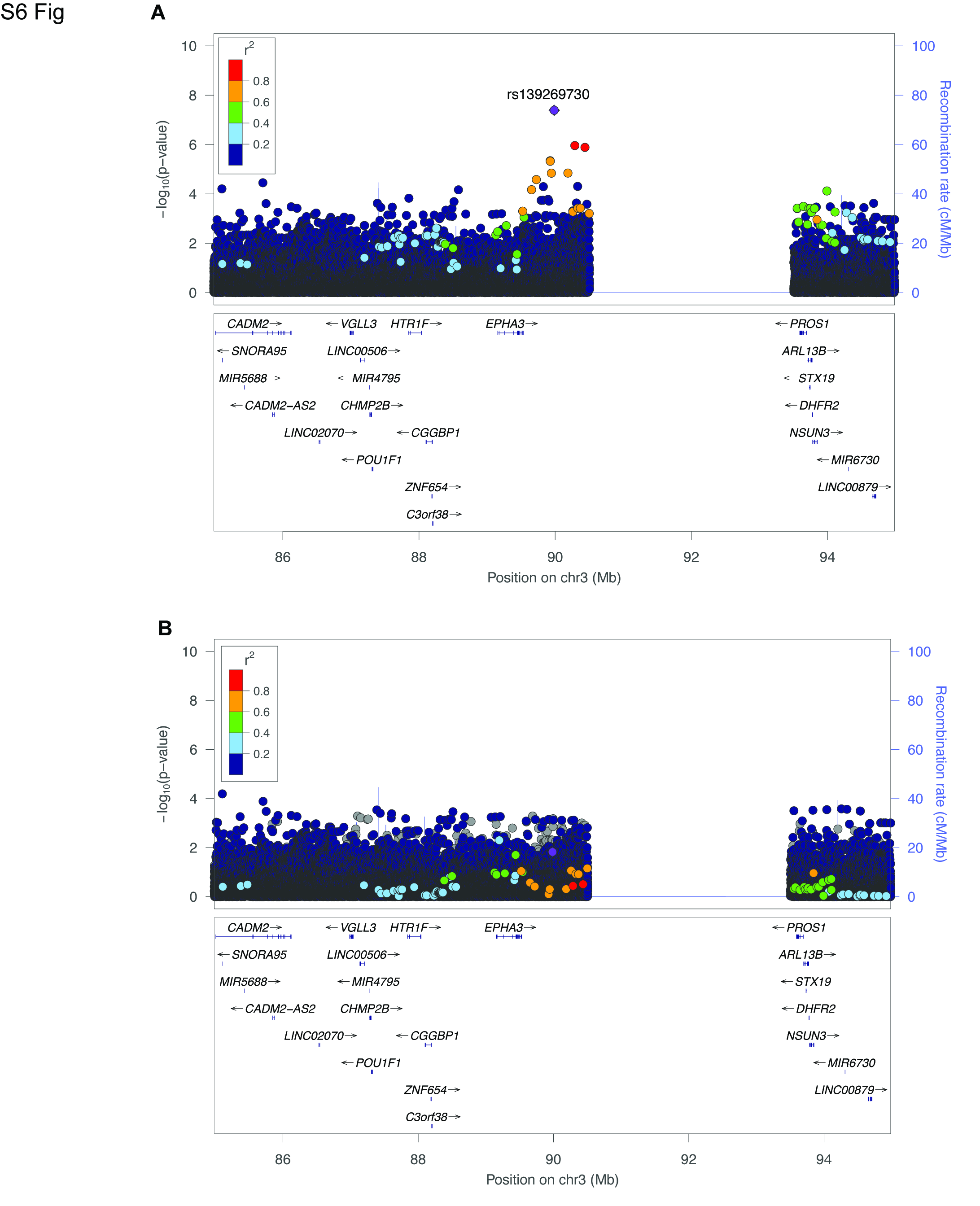

Supplement: S6 Fig — (A) The purple diamond represents rs139269730, the strongest associated variant at this locus (rs139269730-T: β = -0.243, SE = 0.044, P = 4.1x10^-8^, effect allele frequency = 0.74). Other variants are colored based on LD with the lead variant within the METSIM subjects. (B) After conditioning on rs139269730, no additional association signals persisted. The ~3 Mb gap in variants observed in the plots represents the centromere of chromosome 3. The only genome-wide significant variant had moderate imputation quality (r2 = 0.74) and may represent a false positive, thus, this locus was excluded from further analyses. (TIF) [file pgen.1009019.s006.tif]

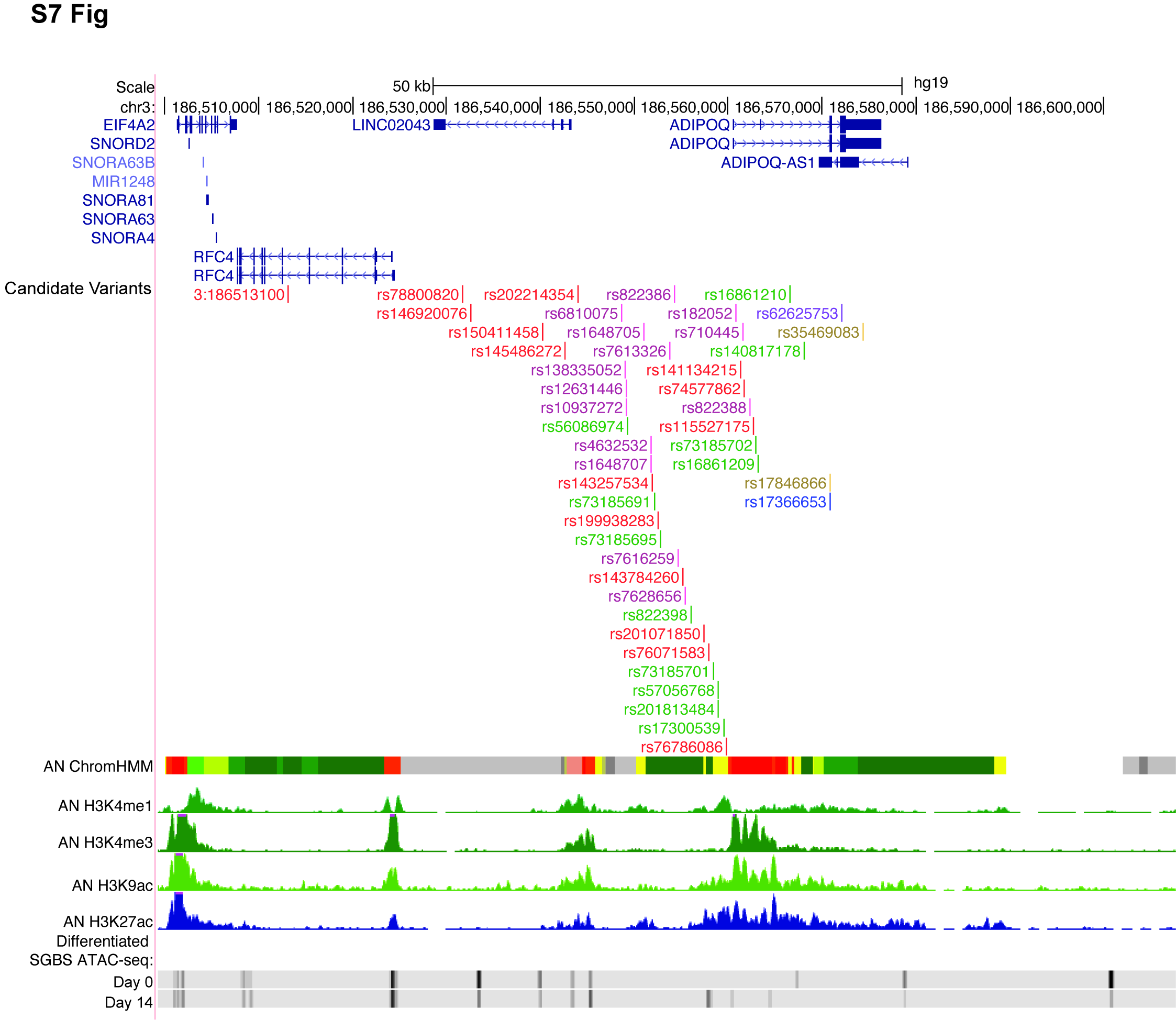

Supplement: S7 Fig — The lead adiponectin-associated variants at ADIPOQ signals A-G (excluding signal ‘D’) and variants in high LD (r2≥0.80) are shown. Variants representing signal ‘A’ are shown in red, signal ‘B’ in magenta, signal ‘C’ in green, signal ‘E’ in navy, signal ‘F’ in brown, and signal ‘G’ in dark purple. (TIF) [file pgen.1009019.s007.tif]

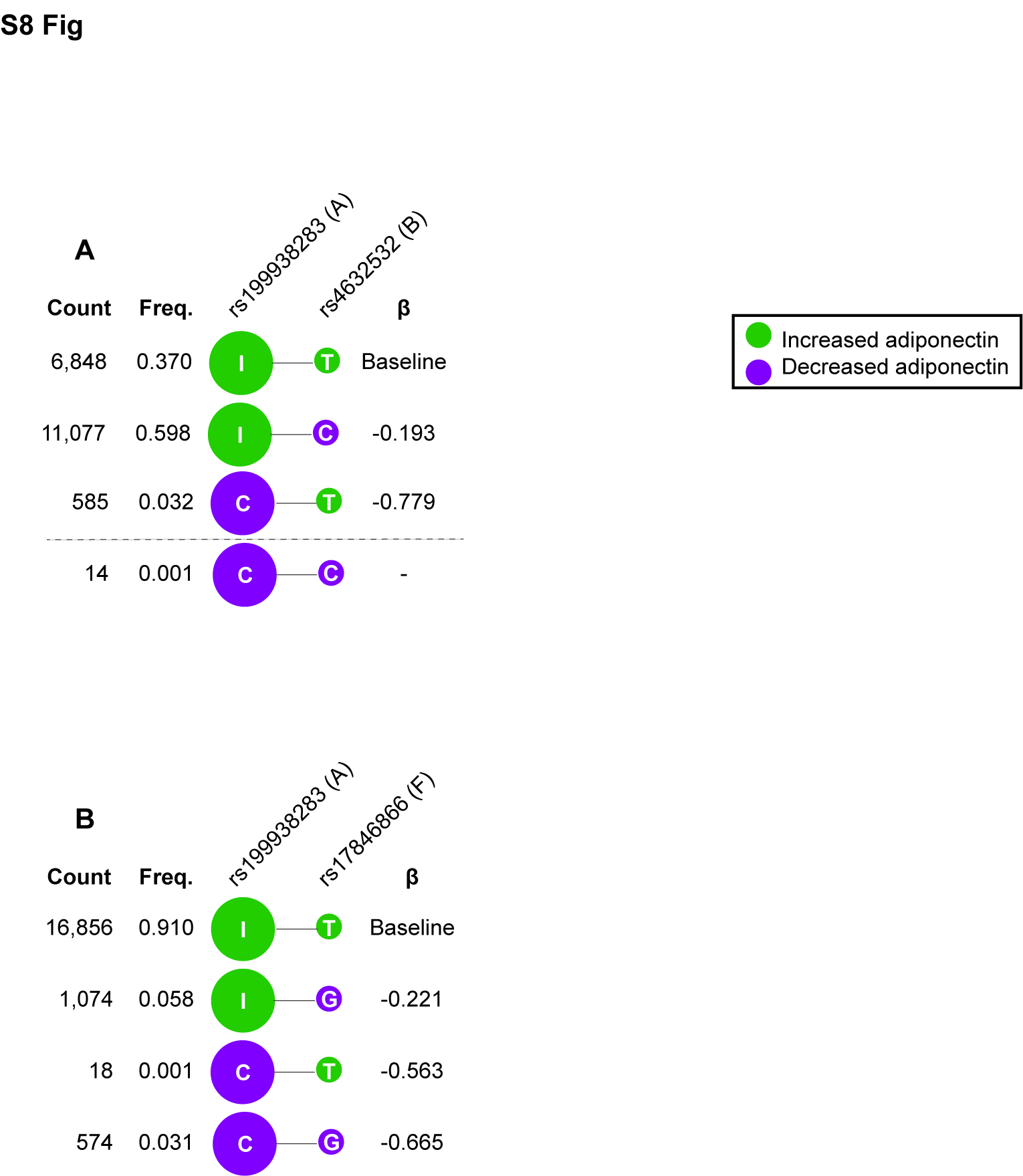

Supplement: S8 Fig — Haplotypes were constructed in 9,262 participants from the METSIM study using the lead variant of each association signal using HaploStats. ‘Count’ indicates the number of estimated haplotypes. (A) Haplotypes for association signals ‘A’ and ‘B’. (B) Haplotypes for association signals ‘A’ and ‘F’. Haplotypes with the same allele for any given signal (e.g. signal ‘A’) show different effect sizes (betas) consistent with their single variant results for alleles at the other signals (e.g. B, F). Alleles associated with lower adiponectin are shown in purple while alleles associated with higher adiponectin are shown in green. Haplotype association was performed with adiponectin inverse normalized residuals after adjusting for age, age2, and BMI using the haplotype containing the adiponectin-increasing alleles at all seven signals as the reference. (TIF) [file pgen.1009019.s008.tif]

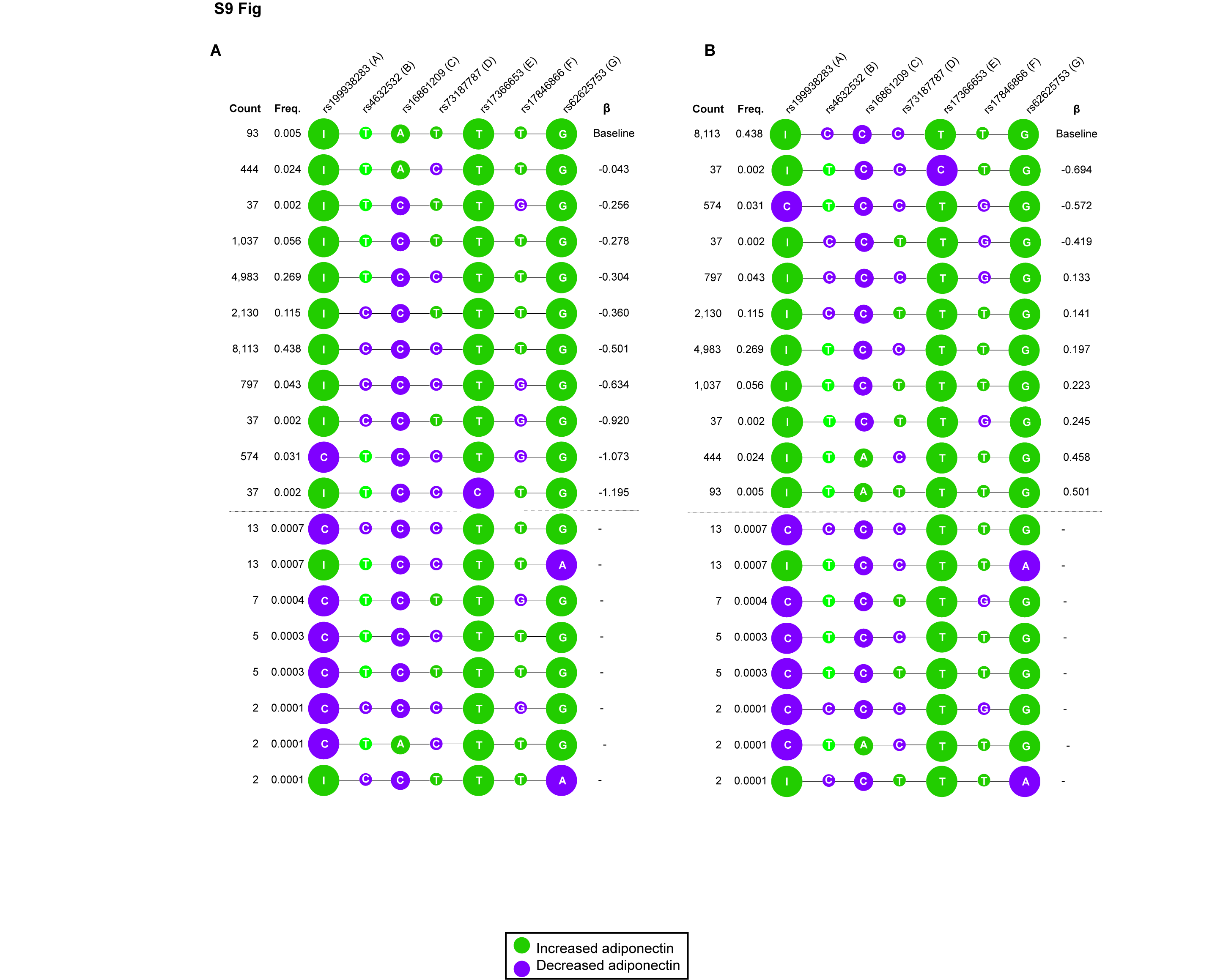

Supplement: S9 Fig — A) Haplotype of all adiponectin-increasing alleles used as the baseline reference. B) Haplotype with the largest sample size used as the baseline reference (same as the baseline reference haplotype used in S10 Fig). Haplotypes with the same allele for any given signal (e.g. signal ‘A’) show different effect sizes (betas) consistent with their single variant results for alleles at the other signals (e.g. B, C, etc). Haplotypes were constructed with the lead variant of each association signal using HaploStats. ‘Count’ indicates the number of estimated haplotypes. Alleles associated with lower adiponectin are shown in purple while alleles associated with higher adiponectin are shown in green. Haplotype association was performed with adiponectin inverse normalized residuals after adjusting for age, age2, and BMI using the haplotype containing the adiponectin-increasing alleles at all seven signals as the reference. The dashed line divides common haplotypes from rare (haplotype frequency <0.001. (TIF) [file pgen.1009019.s009.tif]

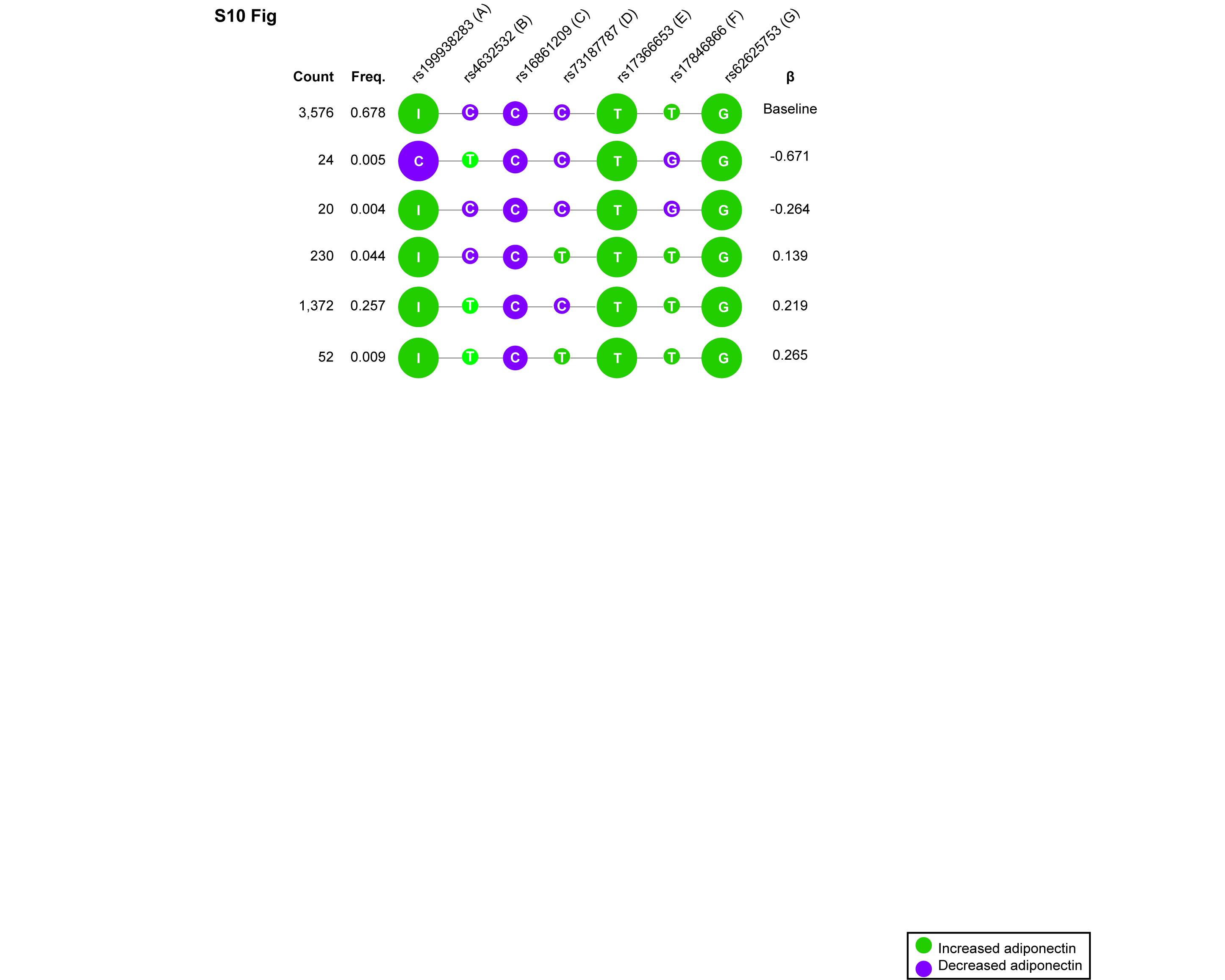

Supplement: S10 Fig — Haplotypes were constructed with the lead variant of each association signal using HaploStats. ‘Count’ indicates the number of estimated haplotypes. Alleles associated with lower adiponectin are shown in purple while alleles associated with higher adiponectin are shown in green. Haplotype association was performed with adiponectin inverse normalized residuals after adjusting for age, age2, and BMI using the most prevalent haplotype as the reference. The order of haplotype effect sizes is consistent with the order of haplotype effect sizes for all 9,262 study participants shown in S9 Fig. (TIF) [file pgen.1009019.s010.tif]

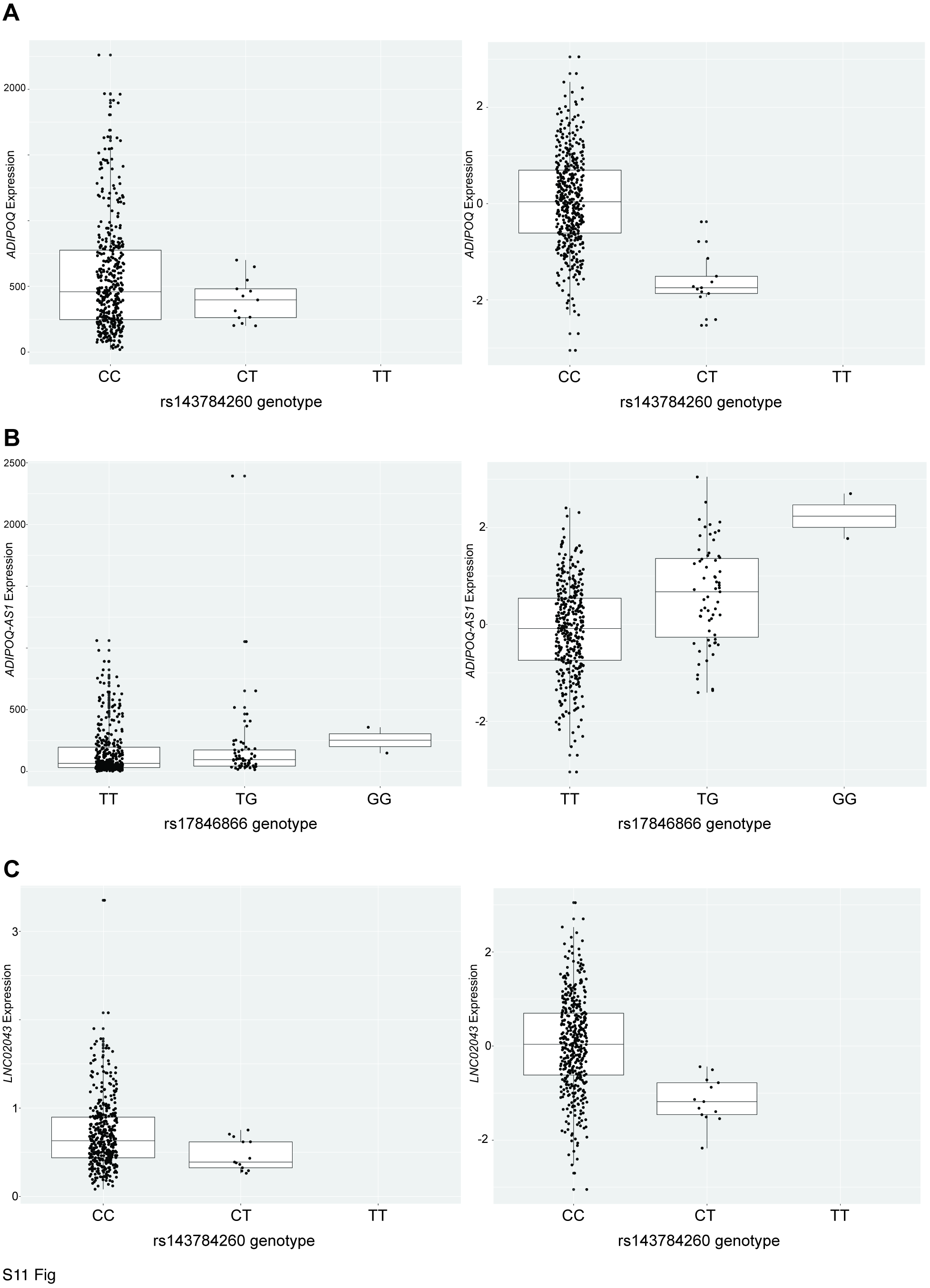

Supplement: S11 Fig — Expression levels are stratified by genotype for the variant most strongly associated with each transcript (eQTL): rs143784260 (A and C) and rs17846866 (B). The lead adiponectin GWAS variant for signal ‘A’, rs199938283, was not available in the eQTL dataset; however, the lead eQTL variant rs143784260 is in perfect LD (r2 = 1.00). For each transcript, expression levels are presented in transcript per million (TPMs) on the left and normalized TPMs after PEER correction on the right. (TIF) [file pgen.1009019.s011.tif]

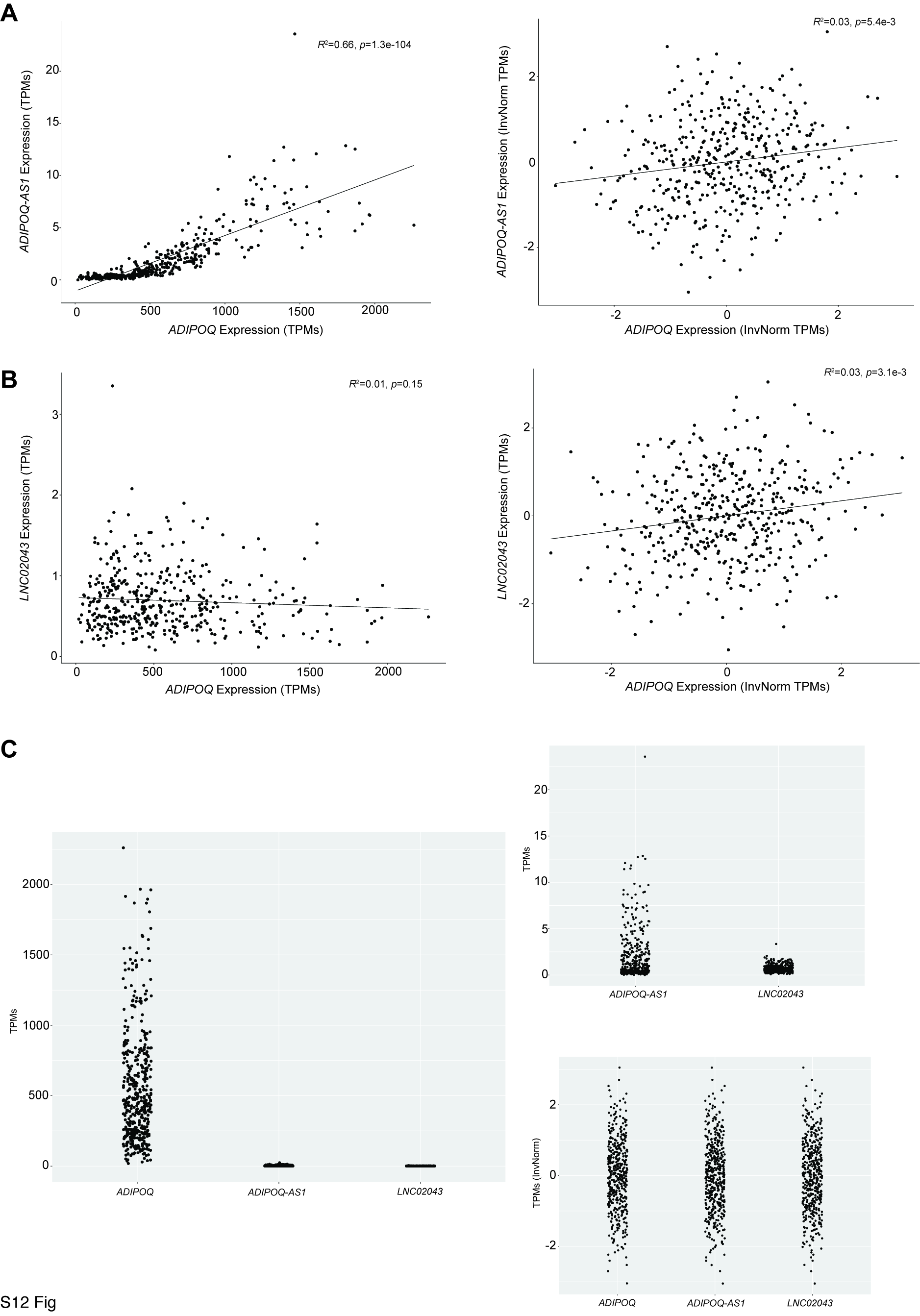

Supplement: S12 Fig — Correlations between expression levels of ADIPOQ and ADIPOQ-AS1 (A) or ADIPOQ and LNC02043 (B) are shown using transcripts per million (TPM) values in the left plot and normalized TPMs after correction for PEER factors on the right. Pearson correlation (R2) values and corresponding P-values are displayed for each plot. (C) Before normalization, the distribution of TPM values is much wider for ADIPOQ (approximate range 0–2500) than for either ADIPOQ-AS1 (approximate range 0–27) or LNC02043 (0–3.5). The upper right plot in (C) is a zoomed-in version of the plot on the left. Following PEER factor correction and inverse normal transformation, TPM values are evenly distributed between the three transcripts (bottom right plot); these values were used in eQTL analyses. (TIF) [file pgen.1009019.s012.tif]

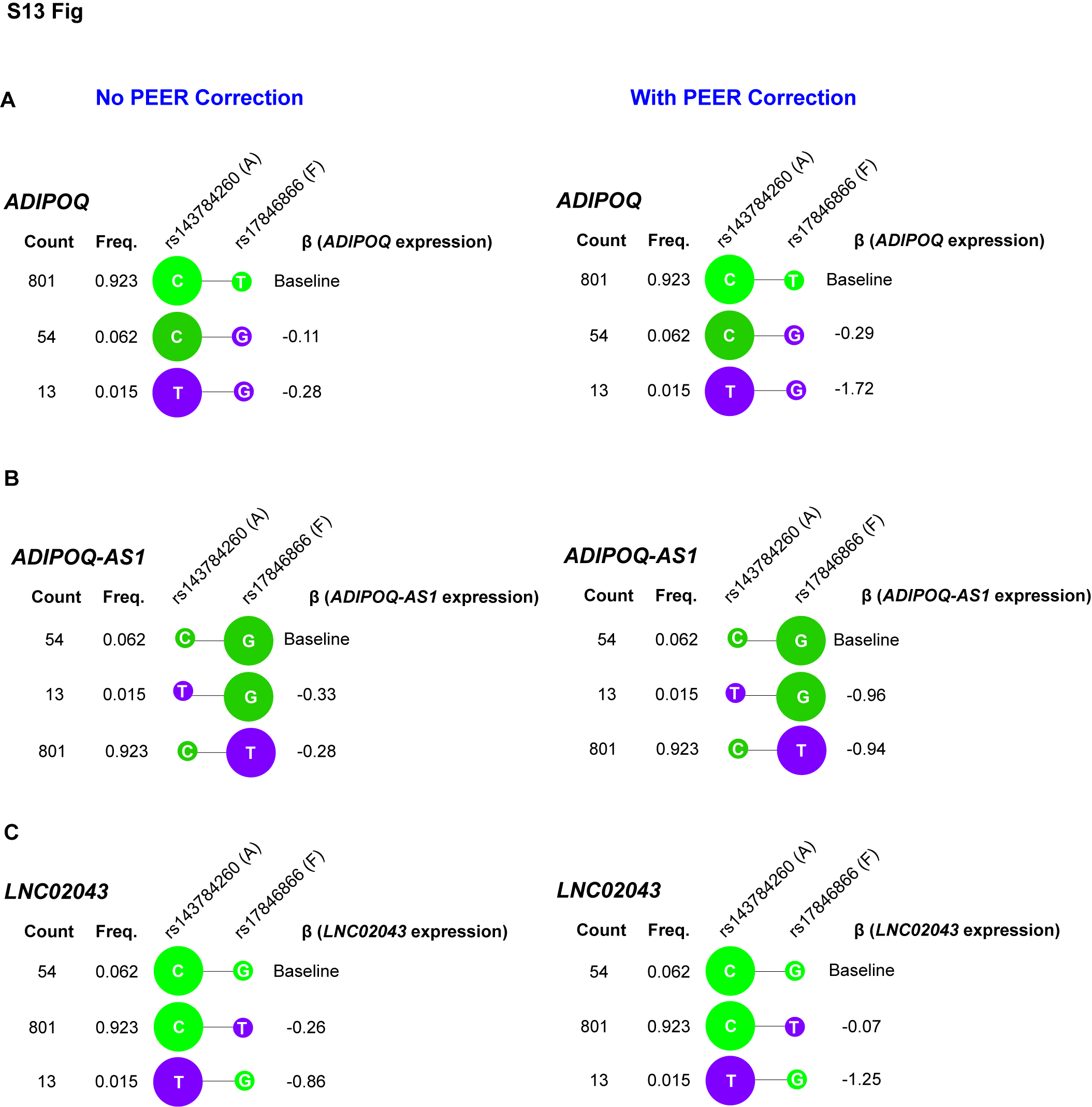

Supplement: S13 Fig — Haplotypes are shown for expression of (A) ADIPOQ, (B) ADIPOQ-AS1, and (C) LNC02043. Expression levels are shown in transcripts per million (TPMs) for plots on the left and normalized TPMs after PEER correction on the right. Haplotypes with the same allele for any given signal (e.g. signal ‘A’) show different effect sizes (betas) consistent with their single variant results for alleles at the other signals (i.e. ‘F’). Haplotypes were constructed with the lead variant of each association signal using HaploStats. ‘Count’ indicates the number of estimated haplotypes. Alleles associated with lower expression are shown in purple, while alleles associated with higher expression are shown in green. (TIF) [file pgen.1009019.s013.tif]

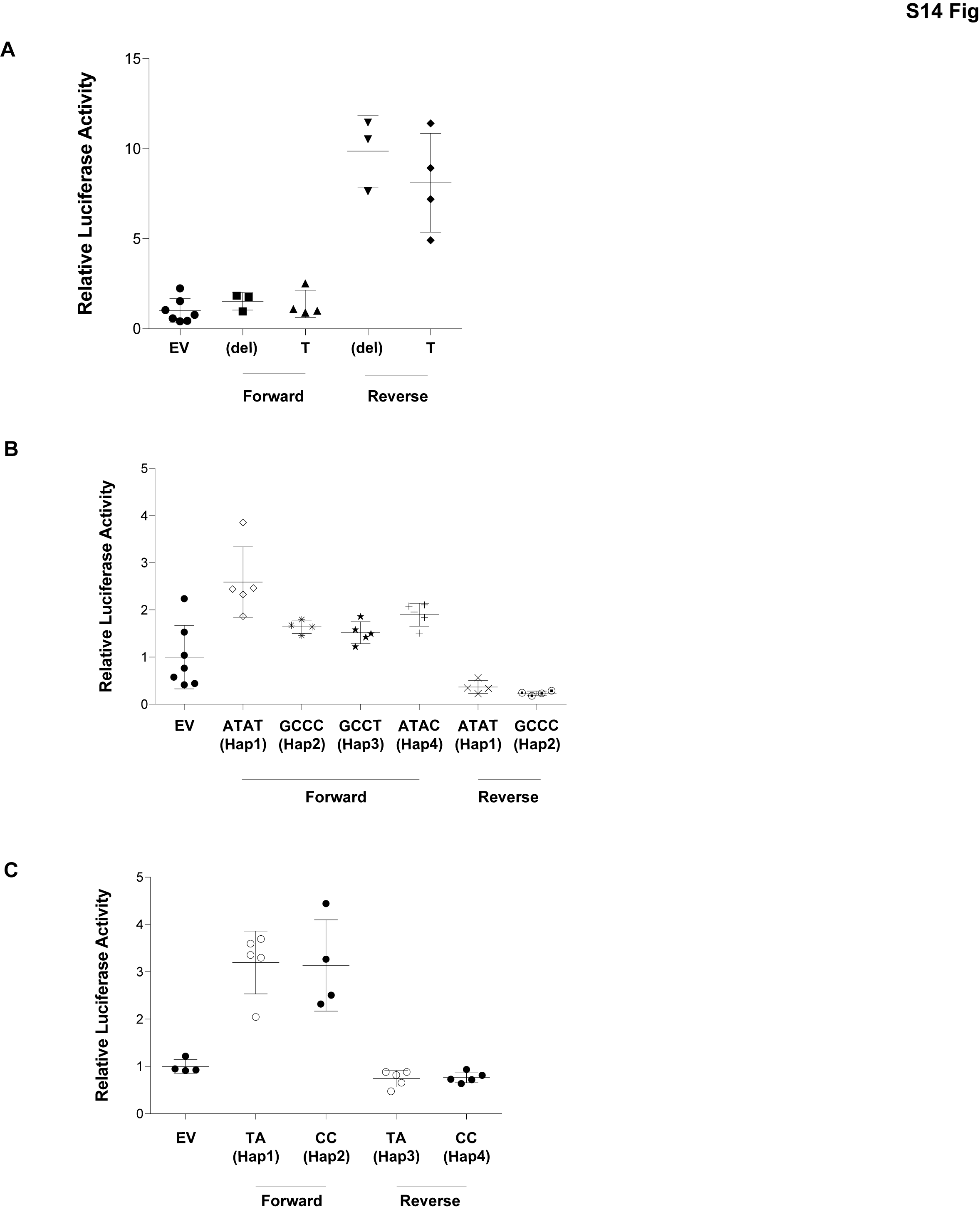

Supplement: S14 Fig — (A) rs150411458 for signal ‘A’. (B) A haplotype of four variants rs1648705 (signal ‘B’), rs4632532 (signal ‘B’), rs1648707 (signal ‘B’), and rs143257534 (signal ‘A’). (C) A haplotype of two variants rs4632532 (signal ‘B’) and rs1648707 (signal ‘B’). (TIF) [file pgen.1009019.s014.tif]

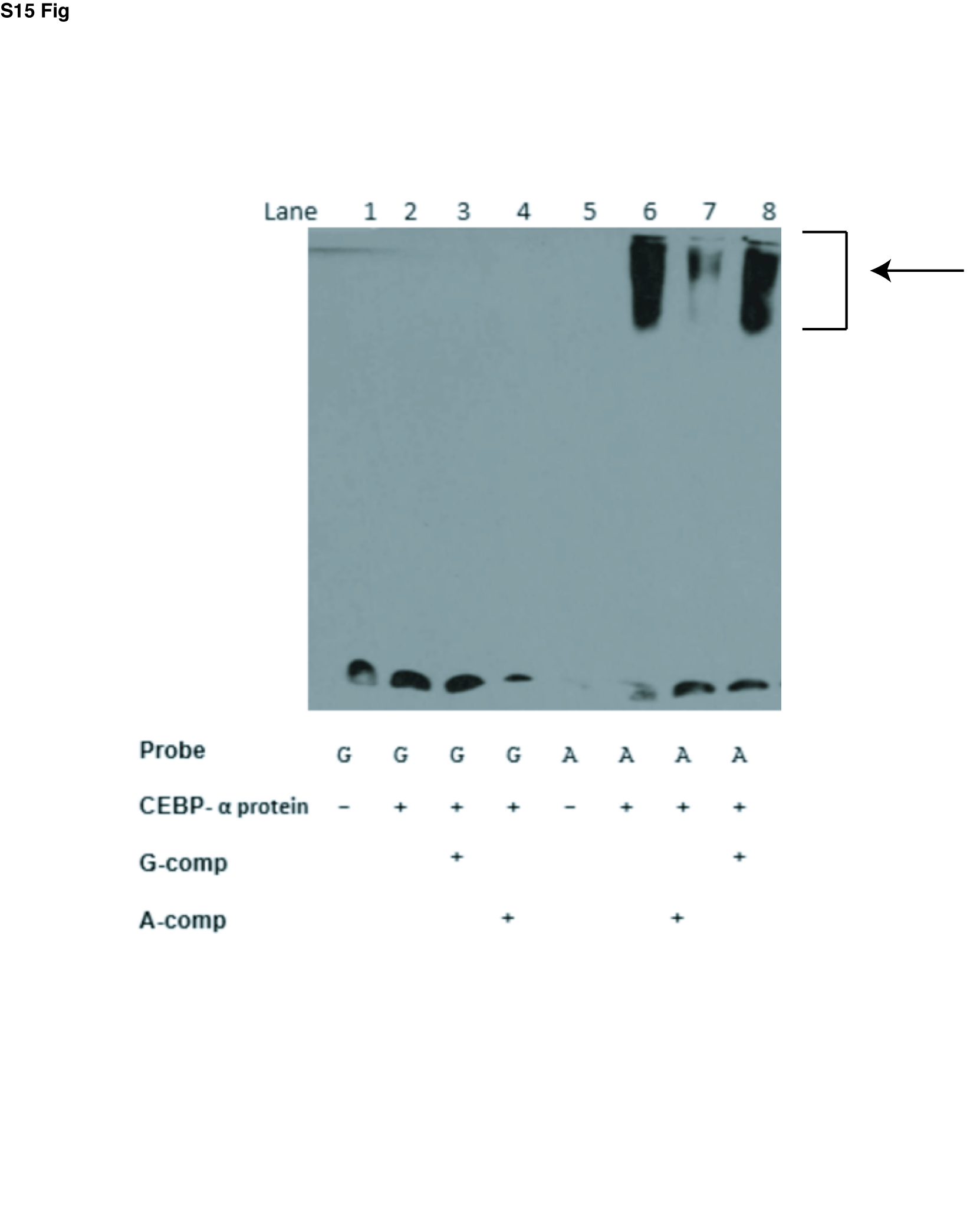

Supplement: S15 Fig — Electromobility shift assay (EMSA) with biotin-labeled probes containing the A or G allele of rs76071583 and purified CEBPA protein show an allele-specific band (lane 6 versus lane 2) that is competed away more effectively by 50-fold excess of unlabeled probe containing the A allele (lane 7) than the G allele (lane 8). An arrow points to an allele-specific protein complex binding to the A allele. (TIF) [file pgen.1009019.s015.tif]

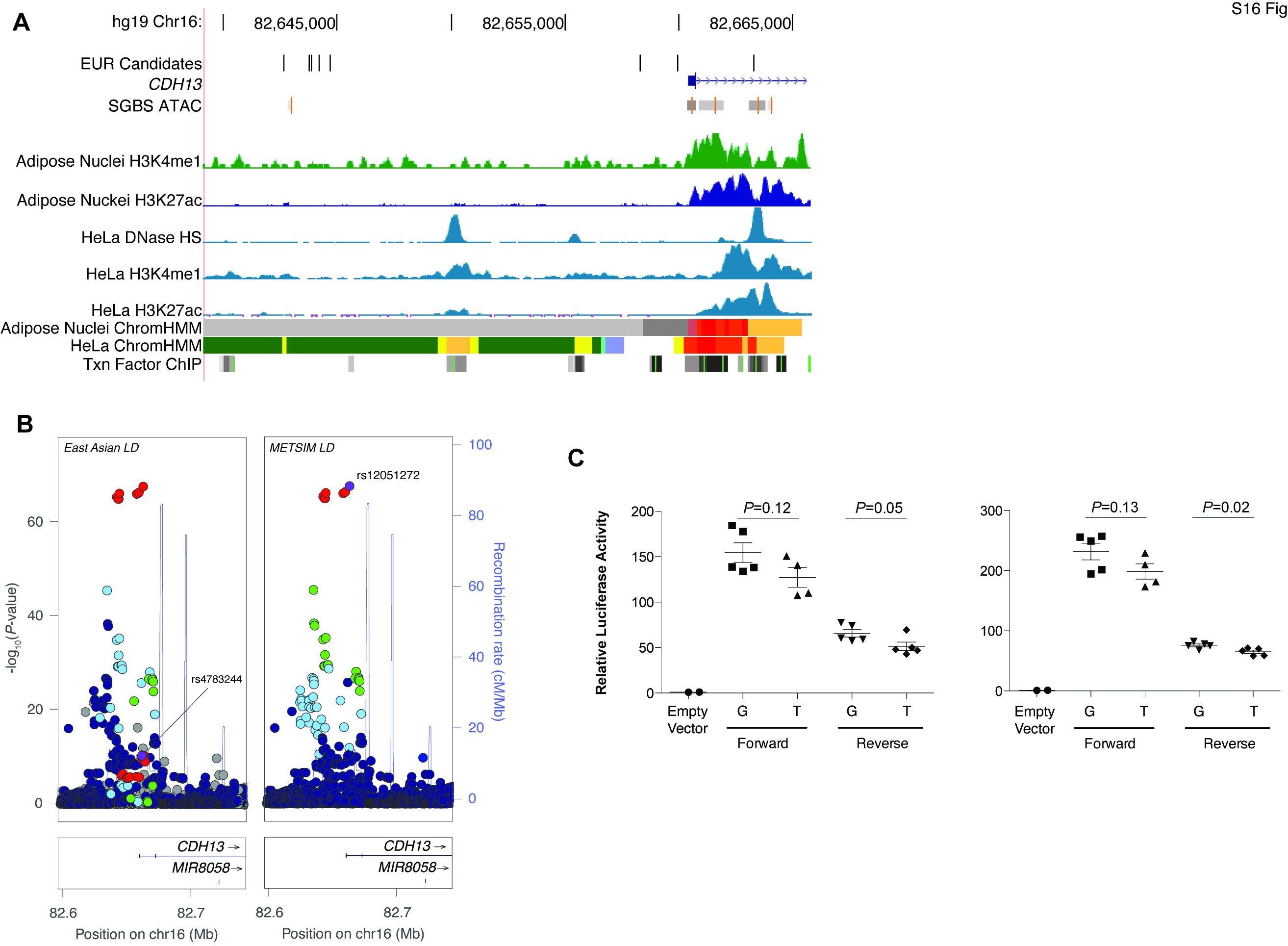

Supplement: S16 Fig — (A) Positions of the lead adiponectin-associated GWAS variant in this study, rs12051272, and seven proxy variants (r2≥0.80; “EUR Candidates”) are shown. (B) Adiponectin genome-wide association results from the METSIM study shown with East Asian LD (1000 Genomes Phase 3) and METSIM LD. The reported lead variant associated with adiponectin in East Asians is rs4783244 is in strong LD with rs12051272 in East Asian populations (r2 = 0.92) but not in Finns(r2 = 0.05). (C) Results from replicated transcriptional activity experiments of CDH13 signal ‘A’ show rs12051272-G is consistently associated with greater transcriptional activity in both the forward and reverse orientations with respect to CDH13 in HeLa cells compared to rs12051272-T and an “empty vector” containing a minimal promoter. Using a linear regression model to analyze data from all three transcriptional activity experiments provides further support that rs12051272-G is associated with greater transcriptional activity in both the forward (P = 0.02) and reverse (P = 0.004) directions. (TIF) [file pgen.1009019.s016.tif]

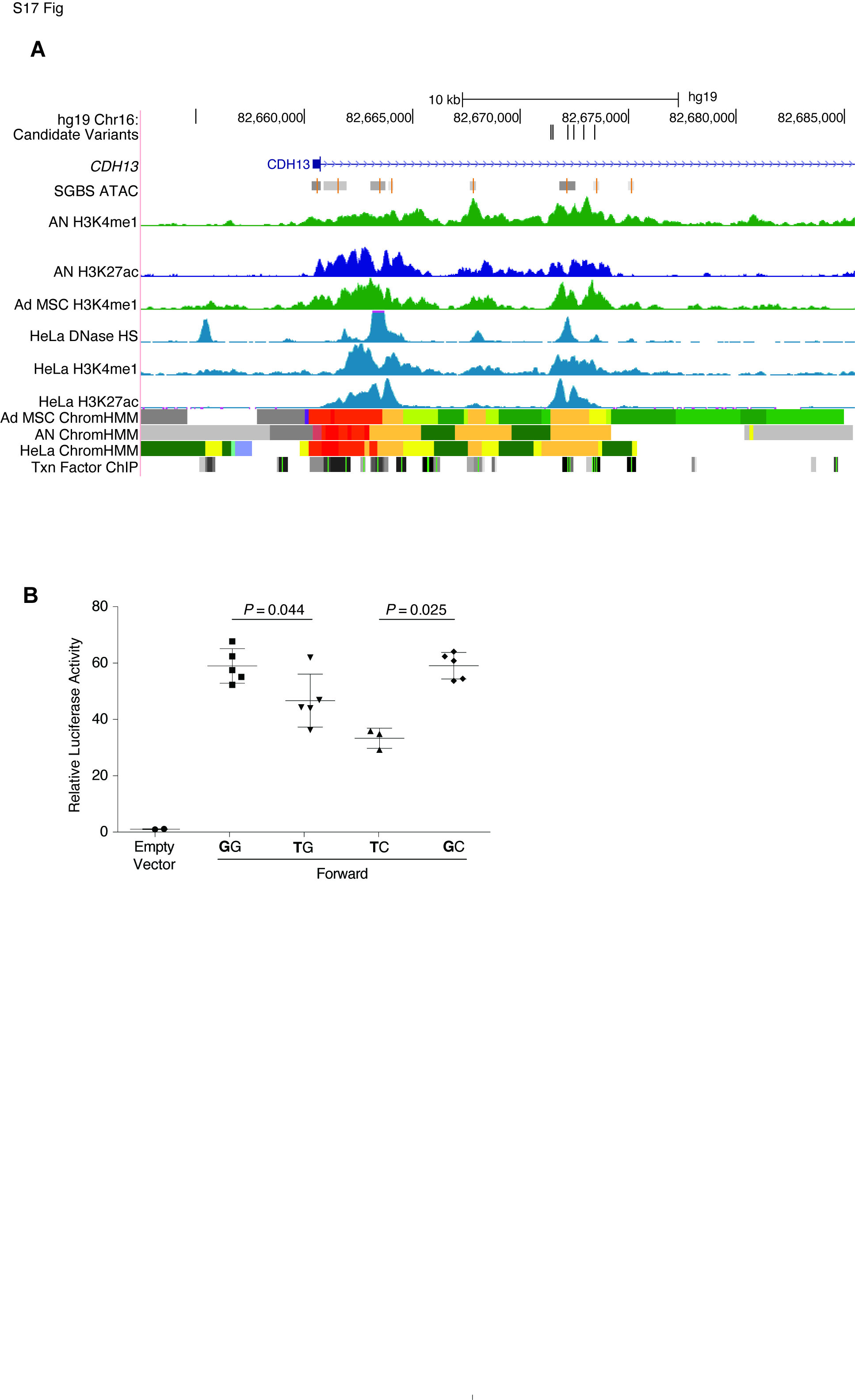

Supplement: S17 Fig — (A) rs4782722 and all five candidate variants in high pairwise LD (r2≥0.80) span a 2.5 kb region in CDH13 introns 1 and 2. (B) At CDH13 signal ‘B’, additional haplotypes of rs4782722 and rs12444113 created by site-directed mutagenesis of rs4782722 implicate rs4782722 as a regulatory variant. Transcriptional reporter assays of a regulatory region spanning rs4782722 and rs12444113 in HeLa cells show that haplotypes containing rs4782722-G (GG and GC) exhibited greater transcriptional activity than haplotypes containing rs4782722-T (TG and TC) in the forward and reverse orientations. Each dot represents transcriptional activity of an independent experimental clone. (TIF) [file pgen.1009019.s017.tif]

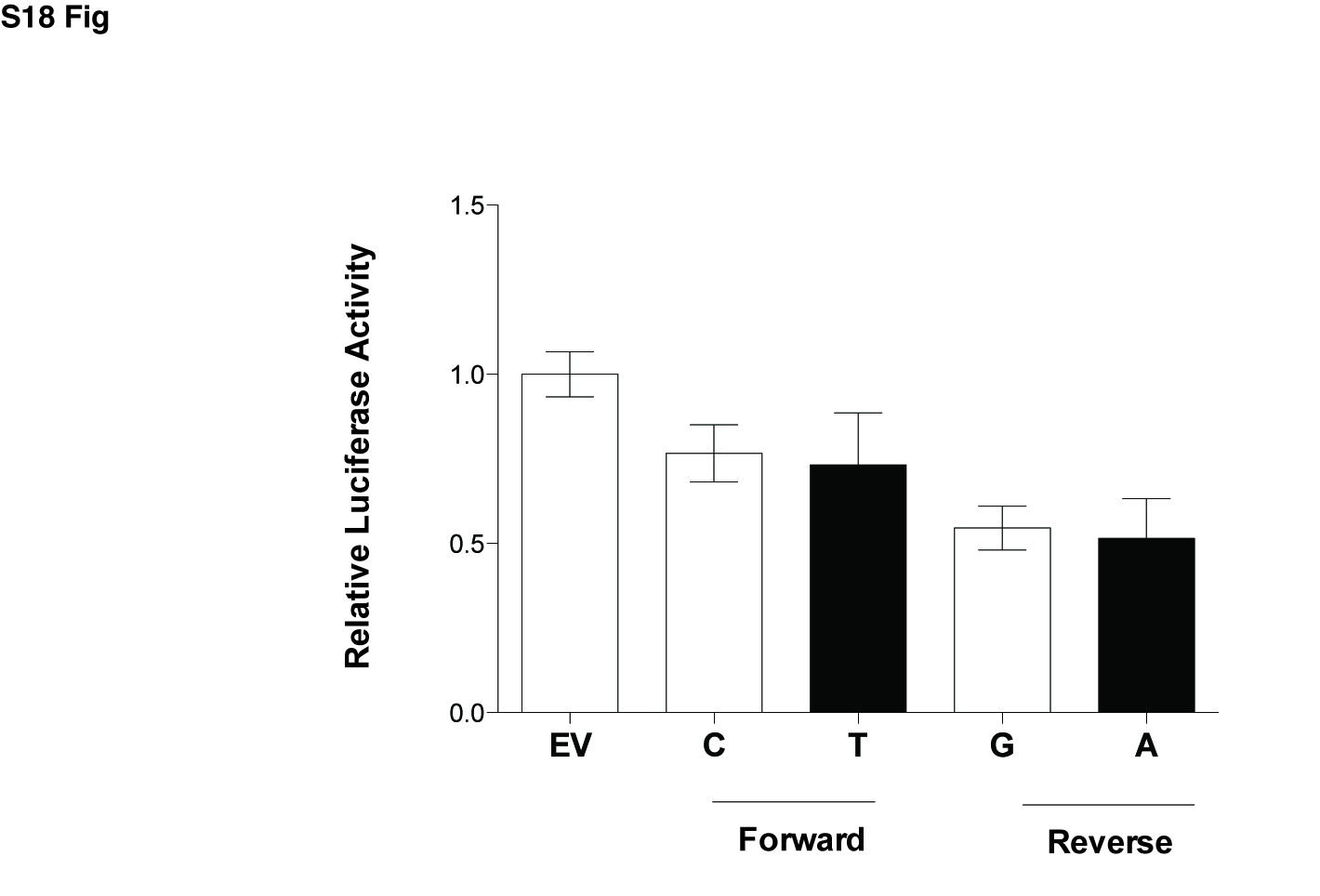

Supplement: S18 Fig — rs3910232 is a proxy of rs4782722 but does not appear to contribute to transcriptional activity differences at this locus. (TIF) [file pgen.1009019.s018.tif]
